# Increasing quality, throughput and speed of sample preparation for strand-specific messenger RNA sequencing

**DOI:** 10.1186/s12864-017-3900-6

**Published:** 2017-07-05

**Authors:** Simon Haile, Richard D. Corbett, Tina MacLeod, Steve Bilobram, Duane Smailus, Philip Tsao, Heather Kirk, Helen McDonald, Pawan Pandoh, Miruna Bala, Martin Hirst, Diane Miller, Richard A. Moore, Andrew J. Mungall, Jacquie Schein, Robin J. Coope, Yussanne Ma, Yongjun Zhao, Rob A. Holt, Steven J. Jones, Marco A. Marra

**Affiliations:** 1 Canada’s Michael Smith Genome Sciences Centre, BC Cancer Agency, 675 West 10th Avenue, Vancouver, BC V5Z 1L3 Canada; 2 Department of Microbiology and Immunology, Michael Smith Laboratories Centre for High-Throughput Biology, University of British Columbia, Vancouver, BC V6T 1Z4 Canada; 3 Department of Medical Genetics, University of British Columbia, Vancouver, BC V6T 1Z3 Canada

**Keywords:** Ampure XP magnetic beads, Next-generation sequencing, Library construction, Strand-specific, dUTP, Uracil DNA N-Glycosylase, Ligation, mRNA, RNA-seq, Illumina, Ligation, Reverse transcriptase

## Abstract

**Background:**

RNA-Sequencing **(**RNA-seq) is now commonly used to reveal quantitative spatiotemporal snapshots of the transcriptome, the structures of transcripts (splice variants and fusions) and landscapes of expressed mutations. However, standard approaches for library construction typically require relatively high amounts of input RNA, are labor intensive, and are time consuming.

**Methods﻿:**

Here, we report the outcome of a systematic effort to optimize and streamline steps in strand-specific RNA-seq library construction.

**Results﻿:**

This work has resulted in the identification of an optimized messenger RNA isolation protocol, a potent reverse transcriptase for cDNA synthesis, and an efficient chemistry and a simplified formulation of library construction reagents. We also present an optimization of bead-based purification and size selection designed to maximize the recovery of cDNA fragments.

**Conclusions:**

These developments have allowed us to assemble a rapid high throughput pipeline that produces high quality data from amounts of total RNA as low as 25 ng. While the focus of this study is on RNA-seq sample preparation, some of these developments are also relevant to other next-generation sequencing library types.

**Electronic supplementary material:**

The online version of this article (doi:10.1186/s12864-017-3900-6) contains supplementary material, which is available to authorized users.

## Background

Revolutionary developments in Next-Generation Sequencing (NGS) technologies and bioinformatics have enabled the pursuit of large scale genomics and functional genomics studies at increasingly reasonable cost and speed. One of the key factors in the generation of high quality NGS data is the process of library construction.

The main purpose of the multi-step library construction process is to provide priming sites and sample-specific indexing barcodes for platform-specific sequencing reactions that are part of various NGS technologies. Typically, this requires ligation of DNA fragments to adapters that contain sequencing primers using T4 DNA ligase. This ligase works optimally on double-stranded substrates [1]. In the case of RNA-seq, this means that double-stranded cDNA needs to be generated a priori. However, a standard double-strand cDNA synthesis would result in strand-agnostic RNA-seq data, which has been shown to have inferior accuracy of transcript quantification and annotation, and is devoid of the capacity to discern anti-sense RNA biology compared to strand-specific RNA-seq (ssRNA-seq) [2–4]. To maintain strand-specific information, a widely employed ssRNA-seq protocol involves incorporation of dUTP during second strand cDNA synthesis [5, 6]. Once the double-stranded cDNA fragments are ligated to adapters, the dUTP-marked strand is selectively destroyed by the enzyme Uracil DNA N-Glycosylase (UNG).

Library construction involves a series of enzymatic reactions, each typically followed by a purification step. A typical library sample preparation for RNA-seq, for example, involves 6–8 such purification steps. Traditionally, these purification steps involved laborious processes such as phenol-chloroform or column-based purifications. The replacement of these purifications with paramagnetic bead-based solid phase reversible immobilization (SPRI) is a significant advance rendering library construction simpler and more amenable to high throughput or robotic handling [7, 8]. The most widely applied and commercialized form of SPRI magnetic beads are Ampure XP beads.

The SPRI technology is based on the reversible binding of nucleic acids to carboxyl-coated paramagnetic beads in the presence of a buffer that contains high salt and polyethylene glycol (PEG). The technology is used in the preferential purification of nucleic acids of a certain size range by adjusting the buffer composition. Reductions in salt and PEG result in selective enrichment of larger fragments. This strategy has been exploited to replace size selection that was traditionally performed through gel-based methods [9, 10].

The vast majority of RNA mass for a given total RNA sample is comprised of a very few species of ribosomal RNAs (rRNAs) [11, 12]. Thus, removal of rRNAs is a common first step in the sample preparation for RNA-seq. Since most rRNAs are not polyadenylated [13], processing of eukaryotic samples involves a positive selection of polyadenylated mRNAs by using the 3′-poly (A) tail as bait [14]. Another approach is a negative selection where rRNAs are depleted directly by using specific probes thereby maintaining informative, non-polyadenylated, non-ribosomal RNAs resulting in a more complete representation of the transcriptome [15–17].

In this study, gel and bead-based size selections are compared and the optimal condition for maximum recovery in bead-based purifications is explored. In addition, the differential behaviour of single versus double stranded DNA/cDNA in bead-based size selection is demonstrated and the implication of this in strand-specific RNA-seq is shown. Other aspects that are addressed in this study include assessment of mRNA isolation protocols, identification of a more robust reverse transcriptase to maximize cDNA yield, evaluation of various library construction kits and optimization of ligation.

## Methods

### RNA and genomic DNA samples

Universal Human Reference (UHR) total RNA was purchased from Stratagene (Catalog #740000). RNA was quantified using Agilent RNA 6000 Nano Kit (Catalog #5067–1511). For some experiments, UHR was spiked with External RNA Controls Consortium (ERCC) spike-in mix from Ambion (Catalog# 4456740). 0.02 μL of the spike-in mix was used per 1 μg UHR total RNA.

Genomic DNA (gDNA) was prepared from Human promyelocytic Leukemia cell line (HL60) using Phenol/Chloroform/Isoamyl Alcohol (25:24:1) phase extraction. gDNA from the tumor samples was prepared using Qiagen’s AllPrep DNA/RNA Mini Kit (Catalog#80204). DNA was quantified using Qubit dsDNA HS DNA assay (Thermo Fisher; Catalog# Q32851).

### mRNA isolation, cDNA synthesis and library construction

The final form of our protocols as well as materials used after optimizations described in this paper are detailed in Additional files 1, 2 and 3 (mRNA isolation, cDNA synthesis, and library construction).

### RNA sequencing and bioinformatic analysis

Most RNA-seq Libraries were sequenced using paired-end 75 base (PE75) sequencing chemistry on HiSeq 2500 instruments following the manufacturer’s protocols (Illumina). In some cases, RNA-seq libraries were sequenced with PE100/125 base reads but were subsequently trimmed to 75 bases prior to alignment-based analysis. gDNA libraries were sequenced at PE125.

 ﻿RNA libraries were aligned against the hg19 reference in concert with Ensembl 61 gene annotations using JAGuaR (*PMID 25062255)*. Resulting BAM files were coordinate-sorted with SAMtools (PMID 19505943) and duplicate reads were marked with Picard (http://broadinstitute.github.io/picard/).

To enable comparisons of metrics that were sensitive to the number of reads used (gene detection and duplicate rates, etc) we randomly down-sampled each of the bams in our comparisons to a consistent number of reads using Picard’s DownsampleSam command. The down-sampled bams were then duplicate read marked with Picard and put through our in-house gene profiling software to estimate the expression of each gene, intron/exon ratios, and transcript coverage bias. In some cases, the down-sampling was done multiple times to ensure the stability of the results to random read sampling.

Base error rates were estimated by counting mismatches in the aligned reads. Using this approach, real Single Nucleotide Variants (SNVs), including Single Nucleotide Polymorphisms (﻿SNPs), would be incorrectly identified as base errors. However, the base error rate (0.002–0.004) was far higher than the SNP frequency rate (0.001) and we are only interested in relative base error frequencies between samples.

Insert size distributions were estimated from read alignments to the mitochondrial genome. This approach provided deep read coverage, avoiding the confounding effects of splicing.

To measure error rates and/or evaluate the quantitative range of transcript levels, we performed ERCC spike-in analyses by re-aligning any read which either unaligned itself, or has an unaligned mate against the ERCC reference. The re-alignments are performed using BWA mem (http://arxiv.org/abs/1303.3997) and requiring a highly specific match (−K 40).

Strand-specificity was estimated by calculating the fraction of Ensembl 61 exon-exon junctions with 0 reads mapping to the anti-sense strand. We focus on reads spanning exon-exon junctions to avoid confounding reads that may originate from genomic DNA background.

## Results and discussion

**Fig. 1:**
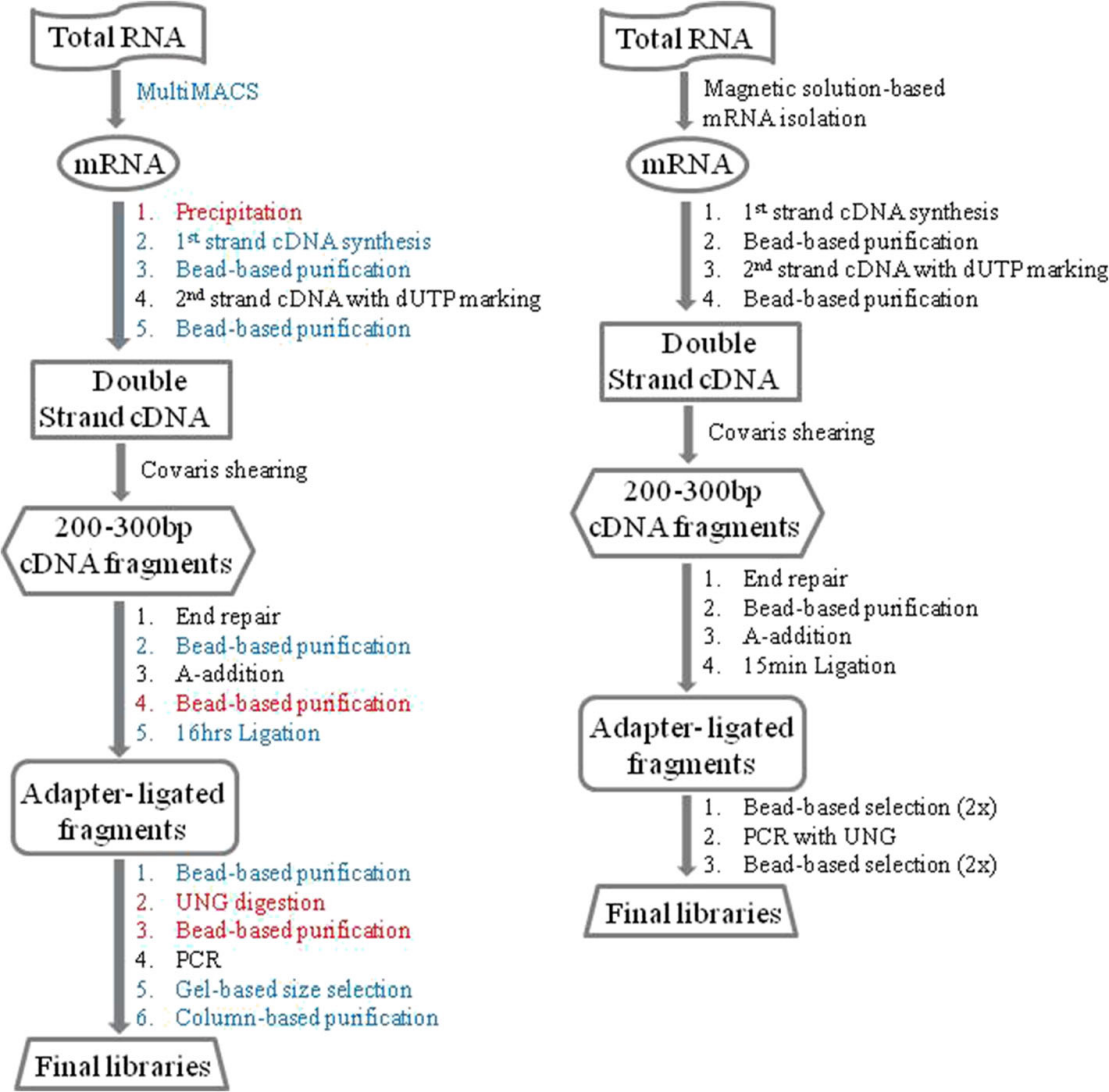
Workflow of ssRNA-seq pipeline at our facility. On the left is the previous version of our pipeline and the on the right is the new version. *Red font* denotes steps which are removed in the new version and blue font represents process or reagent modifications

The focus of this study is the optimization and streamlining of sample preparation for RNA-seq. Our workflow before and after these changes is depicted in Fig. 1. In the following sections, we describe the major aspects of such optimization measures implemented to improve library quality, automate steps in sample preparation, and streamline procedures to shorten the turnaround time at our sequencing facility. Unless otherwise noted, the experiments discussed in this study use Universal Human Reference (UHR) total RNA (Stratagene) as input. UHR RNA is a mixture of RNA prepared from 10 different cancer cell lines to represent the majority of RNA species expressed in various tumors.

Of note, the order in which data is discussed below is different from what is presented in the workflow depicted in Fig. 1 to enable accurate assessment of data.

### Improving cDNA yield

We have generated thousands of RNA-seq libraries using the widely employed reverse transcriptase Superscript II (SSII). We noted a decline in the performance of the enzyme overtime, and observed an inverse correlation between the amount of SSII and cDNA yield with increased yield resulting in better library quality (Additional file 4: Figure S1). This occurred despite the fact that all enzyme amounts used were at <10% of reaction volume, which as a general rule, is the maximum proposition typically used to avoid the inhibitory effect of glycerol in the enzyme storage buffer. We adopted 0.5 μL of enzyme per 25 μL reaction for routine construction of ssRNA–seq libraries and we used this amount in subsequent experiments in this study.

**Fig. 2:**
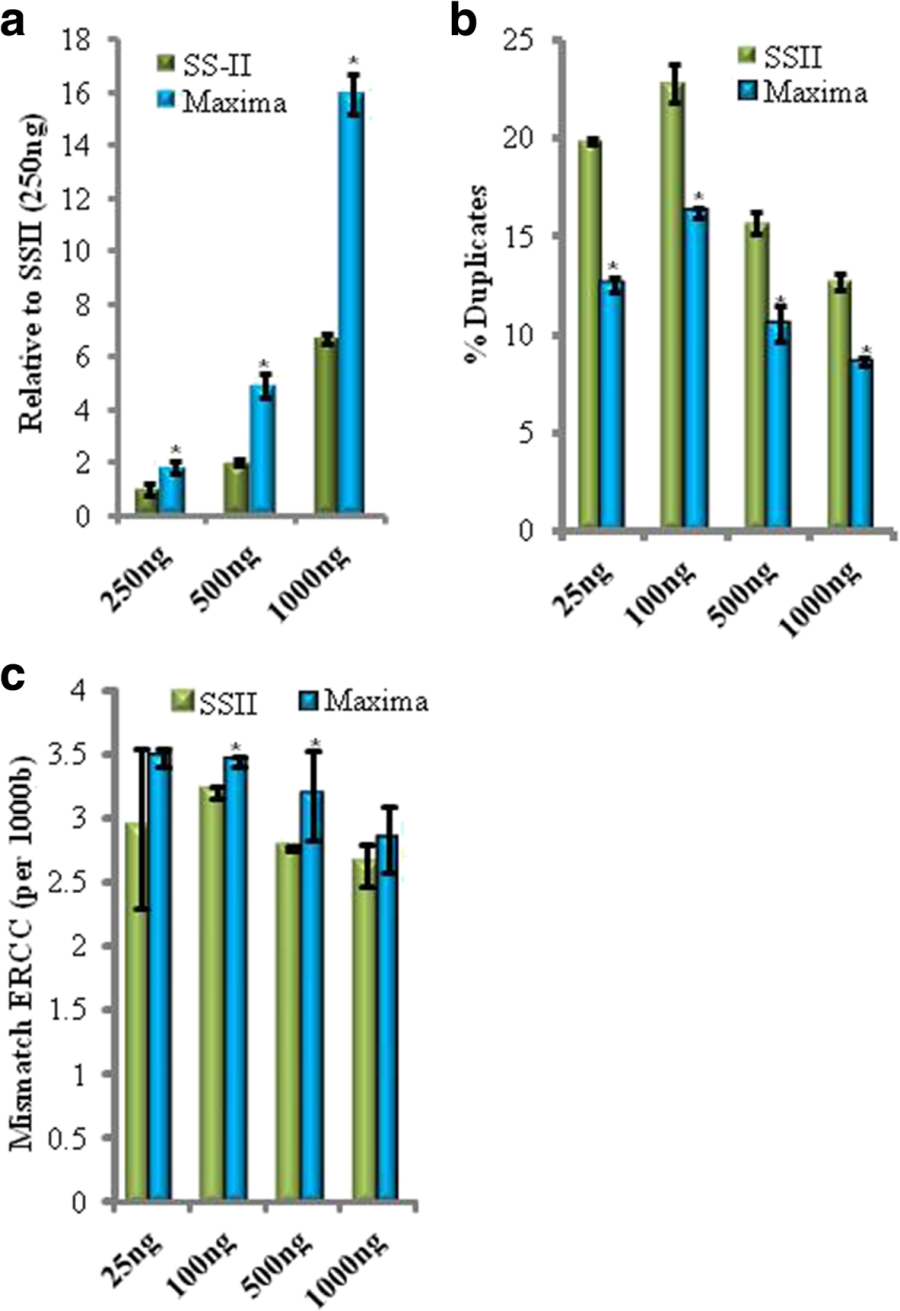
The Maxima H Minus reverse transcriptase provides higher yield of cDNA and quality of libraries. **a** cDNA yield assessment. X-axis indicates various UHR RNA input amounts used for mRNA isolation and cDNA synthesis. Double strand cDNA was measured using the Qubit HS DNA assay. Values from this assay were normalized relative to the value obtained when using Superscript II (RT-II) for the 250 ng input. **b** Diversity of libraries. Libraries were generated from cDNA samples that were prepared using the best performing RT (Maxima) and Superscript II (SS-II). The resulting sequencing data were analyzed for duplicate rates. **c** ERCC spike-in sequence differences. Mismatch rates were calculated by comparing observed sequences and expected sequences from the known spike-in synthetic RNAs. X-axis represents various UHR RNA input amounts used for mRNA isolation and cDNA synthesis. Y-axis is error rate per 1000 nucleotides. *n* = 3; error bars = Standard Deviation. *P < 0.05. *P* values were calculated using Student’s *t*-test (unpaired and equal variance)

The issues observed with the SSII prompted us to look for alternative reverse transcriptases (RTs). Screening of seven RTs from various vendors (data not shown) led to the identification of the Maxima H Minus (Maxima) from Thermo Fisher Scientific as the most potent reverse transcriptase in our reaction conditions. Figure 2a shows that the Maxima enzyme produces higher cDNA yield relative to SSII**.** Further, the percentage of reads aligned for libraries produced using Maxima was higher, or comparable, to those generated by SSII (Additional file 5: Table S1). All major sequence quality metrics indicated that the use of this RT allows the production of higher quality data (Fig. 2b-c; Additional file 5: Table S1). Duplication rate, for example, was reduced ~1.8 fold (Fig. 2b); suggesting increased transcript diversity**.** This trend applied to all input amounts tested between 25 ng and 1000 ng total RNA input.

To assess RT-associated base error rate, we took advantage of the ERCC spike-in RNA mix that was initially developed by the External RNA Controls Consortium [18, 19]. This mix is comprised of 92 individual synthetic RNAs of known sequence and concentration aimed at representing eukaryotic transcripts of varied lengths. The spike-in RNAs are added to the UHR RNA prior to the mRNA isolation step. Sequence divergence of ERCC transcripts observed in our data could, therefore, be attributed to an aggregate intrinsic error rate of the enzymes involved in cDNA synthesis, library construction and sequencing. By making the RT enzyme the only variable, we could assess the fidelity of RT enzymes. We also estimated error rate by looking at the UHR whole transcriptome data where divergence of sequence was measured relative to a compendium of human reference sequences comprised of known single nucleotide polymorphisms (SNPs). Both assessments suggested the degree of fidelity between Superscript II and Maxima H Minus is not significantly different (Fig. 2c; Additional file 6: Figure S2).

### Gel versus bead-based size selection

**Fig. 3:**
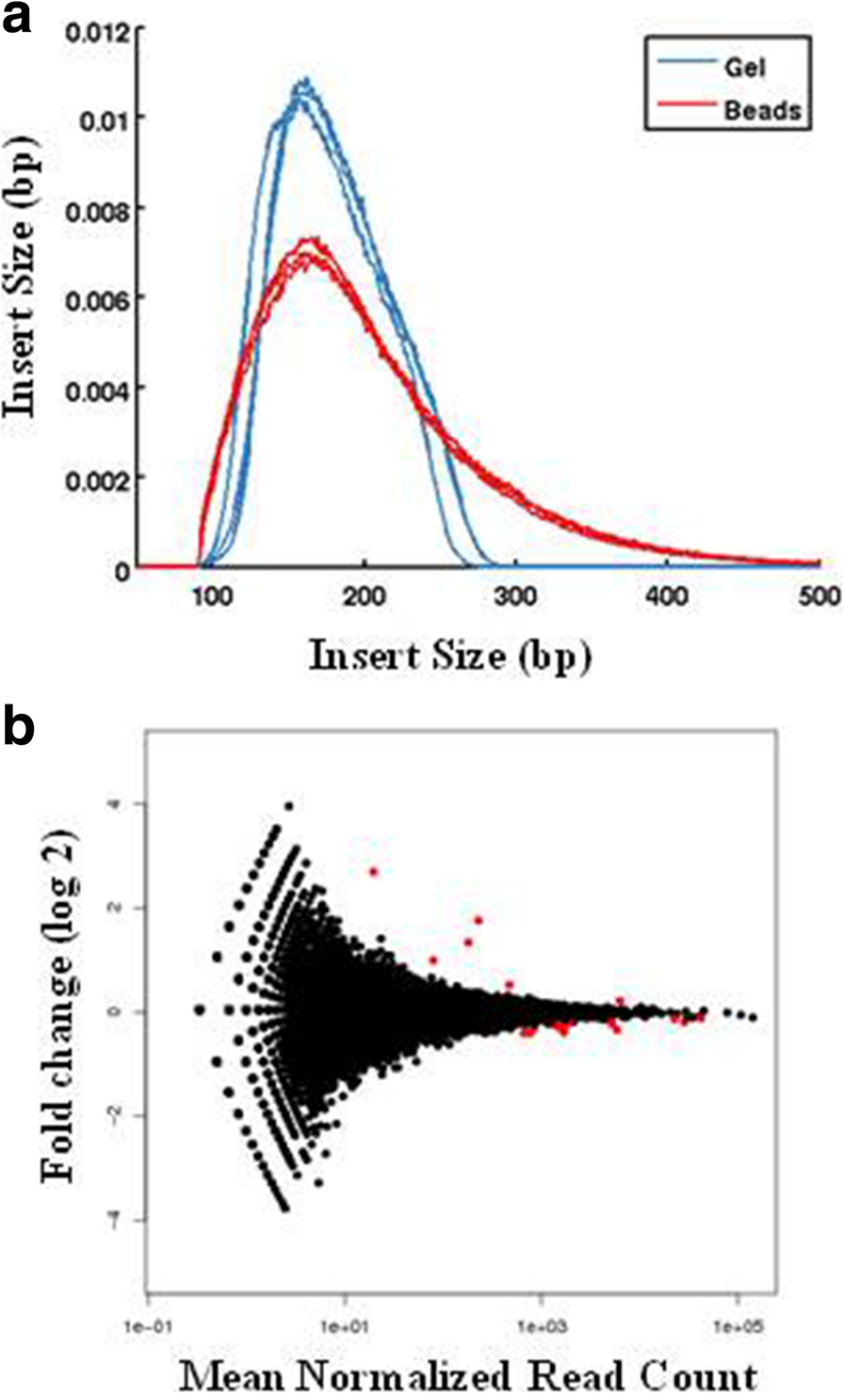
Bead versus gel-based size selection. **a** Insert size. Size profiles were based on reads that mapped to the human mitochondrial genome. **b** Differential gene expression between two conditions. DE-seq plots show genes (*in red dots*) that were differentially expressed at a statistically significant level (FDR ≤ 0.1) as in [21]. *n* = 3 (replicate libraries)

Adapter dimers and unwanted smaller fragments are typically avoided using gel-based size selection, which is a laborious process. We compared bead-based size selection to gel-based size selection in the context of our ssRNA-seq pipeline. The ratio of beads to DNA in the bead-based size selection was 1:1 (on volume to volume basis) and the purification was performed twice. The gel-based size selection targeted 280-400 bp of PCR-enriched library fragments. Library yield was similar between the two conditions (data not shown) despite the broader profile from the bead-based condition (Fig. 3a). This suggested that there is a significant loss of DNA associated with the bead purification, which we address below. Despite these differences, all libraries exceeded the minimum sequencing requirements and were evaluated based on alignment –based sequencing quality metrics (Additional file 5: Table S2). Various sequencing metrics indicated that library quality is comparable between these two conditions (Additional file 5: Table S2; Fig. 3b).

### Optimal bead-DNA binding time is dependent on DNA amount

**Fig. 4:**
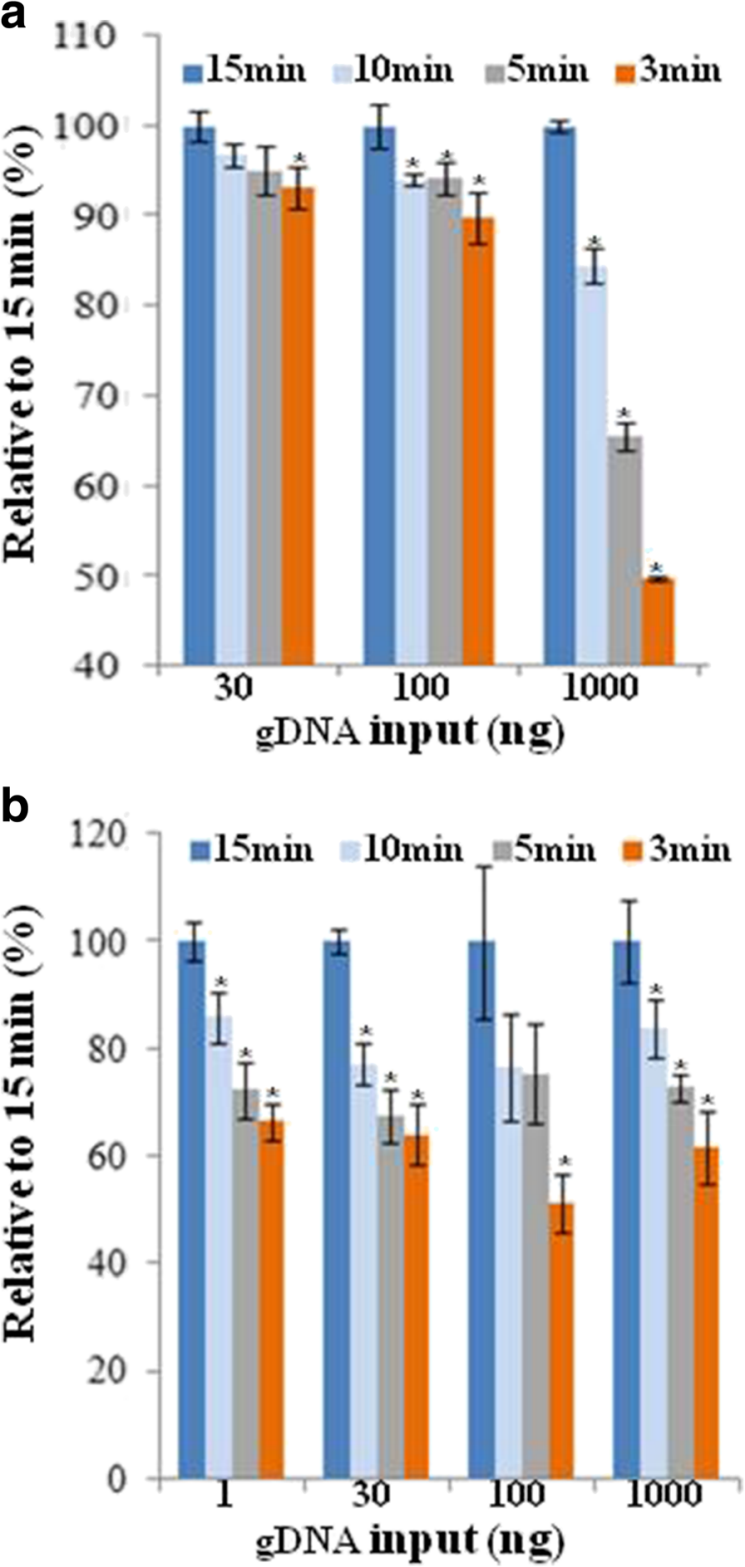
Bead binding time point analysis. **a** Pre-PCR assessment. Various gDNA input amounts (X-axis) were used and libraries were made where the binding time for each of the bead cleanups was varied. The cumulative effect after all cleanups up to the point of post-ligation cleanups is shown. The purified ligated DNA was measured using a Qubit HS assay. The values from this assay were normalized to that of the 1﻿5 min condition. **b** Post-PCR assessment. As in (**a**) but purified DNA was measured after PCR enrichment. i.e. after additional two post-PCR bead-based purifications. *n* = 3; error bars = Standard Deviation. *P < 0.05

The manufacturer specified binding time for Ampure XP beads to DNA is 5 min in the context of the purification of PCR products with 1.8 to 1 beads to DNA ratio. We sought to address whether the optimal binding time varies depending on DNA amount, size and the reaction composition. We chose to investigate this within a typical process that generates PCR-enriched gDNA libraries with a target of 200-250 bp insert size. The generation of such libraries involves 7 purification steps including bead-based size selection. The post-ligation purifications involved two rounds of purifications with 1:1 beads to DNA ratio and further served as size selection steps by removing unwanted small gDNA fragments, excess adapters, and adapter dimers enriching for >200 bp fragments. Similarly, the post-PCR purifications involved two rounds of purifications with a 1:1 ratio of beads to DNA. The pre-ligation steps were performed with a 2:1 ratio so that maximum recovery of DNA was achieved. gDNA input amounts for library construction were 1 ng, 30 ng, 100 ng and 1000 ng and bead binding time points evaluated were 3 min, 5 min, 10 min, and 15 min. The cumulative effect on all the purifications up to the second round of post-ligation purification (Fig. 4a) and up to the second round of post-PCR purification (Fig. 4b) was assessed. A fifteen minute binding time was found to be the most optimal for all input amounts for both the pre-PCR and post-PCR assessments. In the case of the pre-PCR assessment, the difference was statistically significant (*p* < 0.05) but marginal for input amounts up to 100 ng in contrast to the 1000 ng input amount where 15 min binding gave ~2-fold higher recovery relative to the 3 min binding time point. This suggests that higher DNA amounts require a longer binding time to achieve maximum recovery. The number of PCR cycles was adjusted considering input amounts and the PCR products were in the microgram range for all input amounts. Consistent with the pre-PCR data for the 1000 ng input, the post-PCR data also showed a similar trend where 15 min binding resulted in ~1.6 to 2-fold higher recovery relative to the 3 min binding time point for all input amounts. The trends shown in Fig. 4 were also observed for larger DNA fragments and they persist irrespective of reaction composition (data not shown). We also compared the starting DNA amount with the amount recovered after a single purification in the context of the post-PCR purification and found that recovery rate was at ~95% for the 15 min binding time (data not shown). The empirical data presented here shows that recovery of DNA is enhanced by longer incubation times.

### Bead-based purification/size selection after UNG digestion

**Fig. 5:**
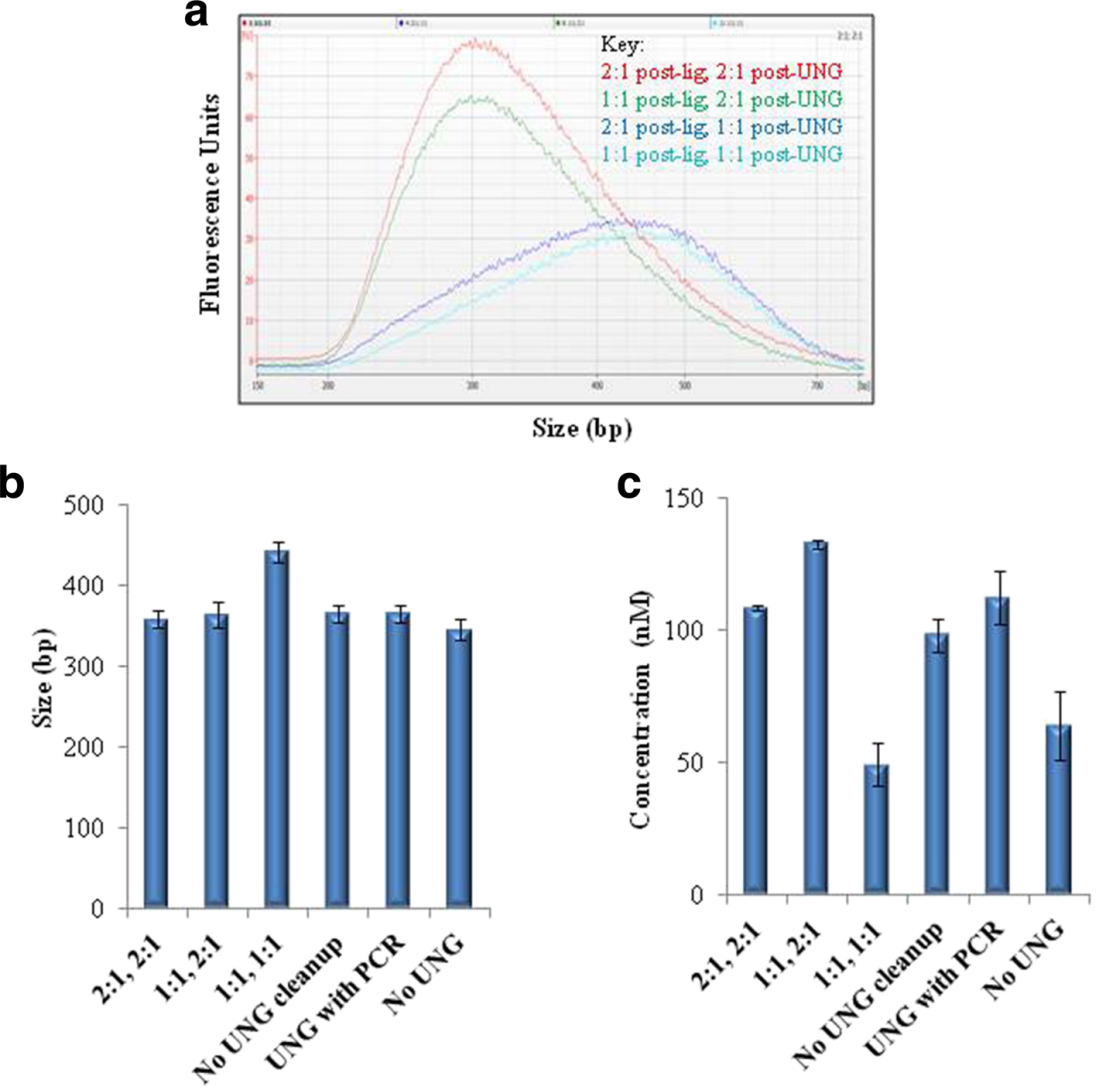
Post-UNG bead-based purifications: library yield data. **a** Effect of bead to DNA ratio on library sizes. Various combinations of 1:1 and 2:1 bead to DNA ratio were applied for post-ligation and post-UNG purifications. The final purified PCR product was run on Agilent DNA 1000 for size profiling. **b** Size profiles of libraries made using variations of the UNG step. The first three conditions where bead amount was varied for post-ligation and post-UNG purifications involve a distinct UNG step. The fourth condition also has a separate UNG step but the reaction is used as a template for PCR without purification in between. The next condition combines UNG and PCR reactions where as the last condition omits the UNG treatment all together. The sizes of these libraries were calculated from fragment smear analysis using Agilent’s software. Input was 1μg UHR t﻿otal RNA. **c** Yield comparison of libraries made using various formats of the UNG step. As in (**b**) but endpoint data is concentration of the final libraries. *n* = 3; error bars = Standard Deviation

For post-ligation purifications, two rounds of purifications with 1:1 ratio are commonly applied for maximal removal of adapter artifacts (mainly dimers)**.** The first purification step is inefficient in removing the unwanted fragments as the ligation buffer contains very high amount of PEG. Additional file 7: Figure S3A shows the final library profiles relative to the sheared material for the gDNA protocol. As discussed above, the strand-specific RNA sequencing protocol that is widely employed involves the dUTP marking and subsequent destruction of the second strand cDNA via UNG [5, 6]. To be able to have a streamlined workflow, we first aimed at splitting the two rounds of purifications with one round being after ligation and the other after UNG digestion. The final library profiles relative to the sheared material for ssRNA-seq are shown in Additional file 7: Figure S3B. Relative to the gDNA library profiles, we observed that the final libraries from the ssRNA-seq protocol were larger in size despite the profiles of the sheared material being similar. These data suggested that the 1:1 purification might be resulting in removal of relatively larger fragments when applied after UNG digestion. To directly demonstrate this, we looked at various combinations of 1:1 versus 2:1 ratios for both post-ligation and post-UNG purification steps. As shown in Fig. 5a, both conditions where we observed larger library sizes involve 1:1 post-UNG. There was no significant difference in size between 1:1 and 2:1 post-ligation when post-UNG was held constant at 2:1. Importantly, libraries were larger in 1:1 post-UNG versus 2:1 post-UNG when post-ligation was held constant at 2:1. These data confirm that purification with 1:1 beads to DNA ratio is not optimal for purification after UNG digestion.

### Identification of an optimal UNG reaction protocol

One way to avoid 1:1 post-UNG purification is to perform two rounds of purifications with 1:1 ratio post-ligation and 2:1 ratio post-UNG. However, this adds an additional bead purification step. Alternatives that avoid this extra step include: (a) using the UNG reaction as a template for PCR without purification in between; (b) including the UNG in the reaction mix for PCR and (c) omitting the UNG enzyme all together. A potential caveat for (a) is that impurity might compromise PCR efficiency. Additional concern for (b) relates to the upstream reaction and deactivation incubations for the UNG reaction that might compromise PCR efficiency. The theoretical expectation for (c) to work and still achieve strand-specificity is the inability of the high fidelity PCR enzymes (Phusion in our case) to amplify dU containing template. The extent of background amplification might, however, be too high to achieve acceptable strand-specificity. We evaluated these conditions in parallel at the levels of library yield, library size profiles and sequencing data.

**Fig. 6:**
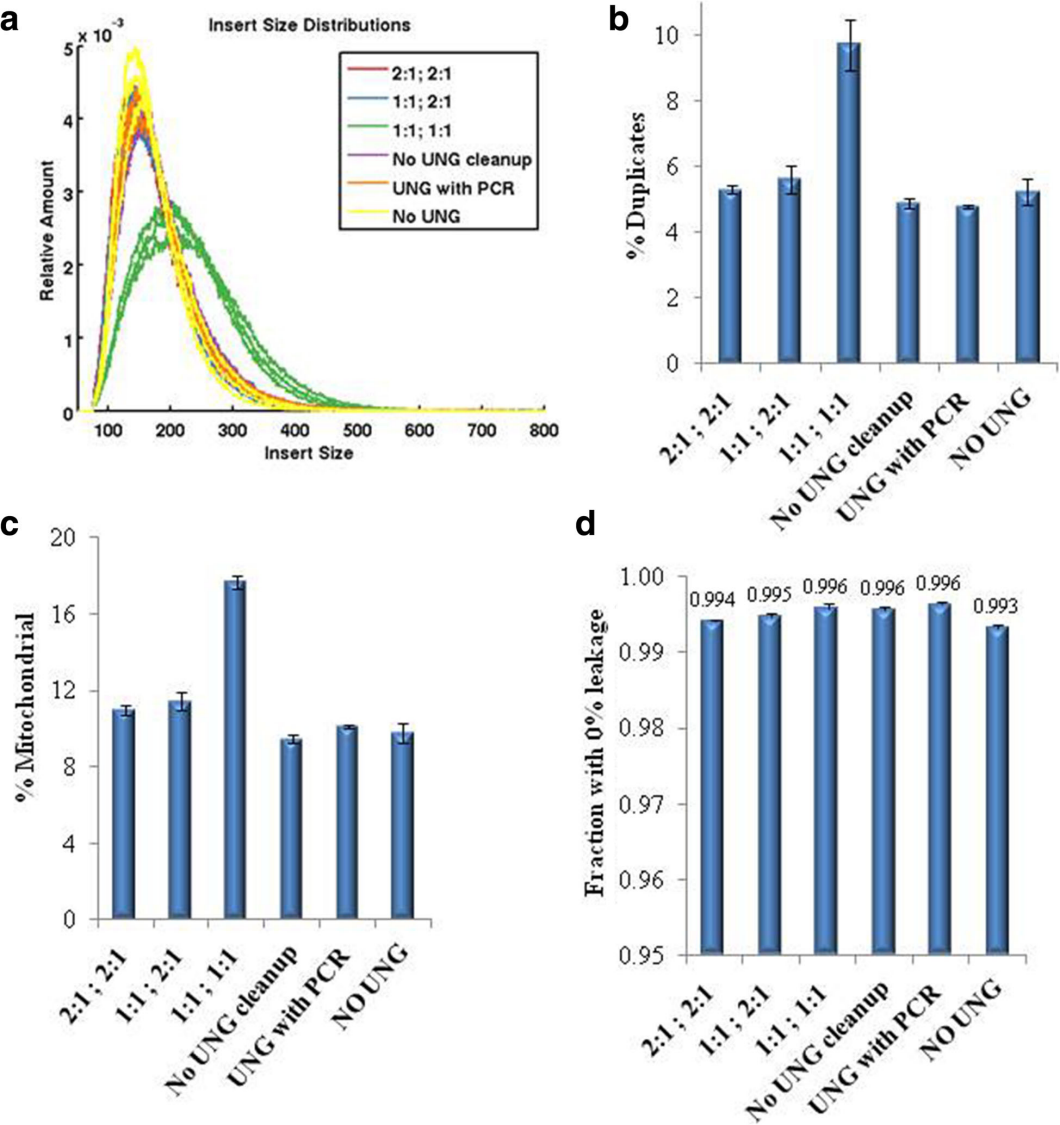
Post-UNG bead-based purifications: Sequencing data. **a** Bioinformatic insert size profiles correlate with lab level data. The same libraries described in 5B and 5C were sequenced. Other post-alignment assessments included % duplicates (**b**), % Mitochondrial (**c**) and s﻿trand-﻿ specificity (**d**). *n* = 3; error bars = Standard Deviation

As expected, all three conditions displayed similar library sizes to those where distinct UNG reaction was applied and 2:1 post-UNG purification was performed (Fig. 5b). Of note, the 1:1 post-UNG condition had largest library size (Fig. 5b) and lowest library yield (Fig. 5c). The next lowest yield was unexpectedly from the condition where UNG was excluded. The decrease in yield was even more severe for this condition upon further increase of the amount of template for PCR (Additional file 8: Figure S4). The other conditions had comparable yield to each other. Insert size was estimated bioinformatically based on mitochondrial transcripts. Consistent with the electrofluorogram data, the 1:1 post-UNG condition had the largest insert size whereas the others were comparable to each other (Fig. 6a). To compare the quality of these libraries, diversity was inferred from duplicate rates (Fig. 6b). Again, the 1:1 post-UNG condition stood out with highest duplicate rate and the others were comparable to each other. A similar trend was observed for the percentage of reads aligning to mitochondria (Fig. 6c). All libraries had >99% strand-specificity even though the condition where UNG was omitted had lower strand-specificity by ~0.3% (Fig. 6d). In contrast, strand-specificity was calculated to be 2.5% for strand-agnostic libraries (Additional file 9: Figure S5). These results collectively suggest that the inclusion of UNG in PCR is the most optimal among the conditions tested.

### Comparison of various library construction kits

**Fig. 7:**
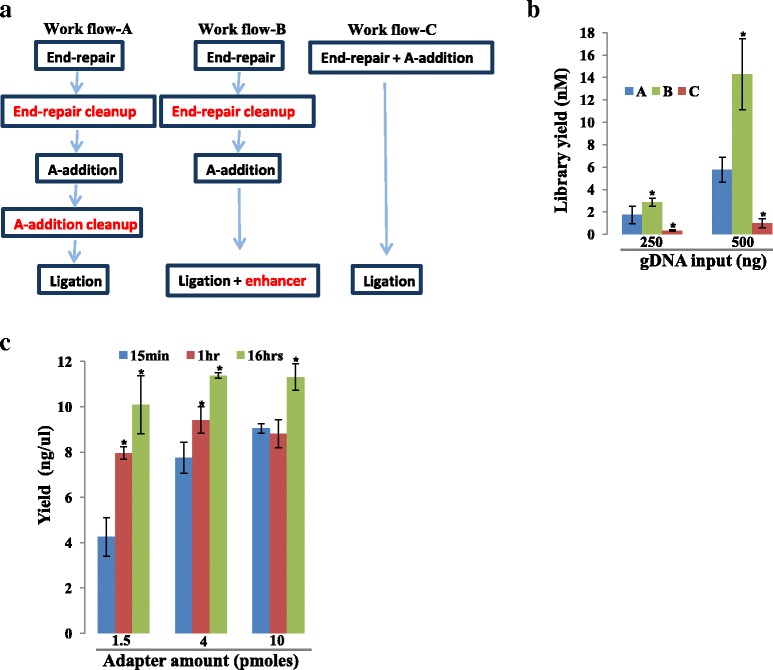
Identification of optimal library construction chemistry and ligation condition. **a** Workflows of three categories of library construction chemistries. Work flow-A has cleanups after every step of library construction and in Work flow-B the cleanup after A-addition is removed where as in the most-streamlined Work flow-C end-repair and A-addition are coupled into one reaction and the cleanup after A-addition is removed. **b** Comparison of library yield between the three NEB workflows. PCR-free libraries were generated using the chemistries that represented the workflows depicted in (**a**) using two different amounts of gDNA as inputs. qPCR was applied to measure the final library yield. **c** Optimization of ligation. For the best performing chemistry (NEB workflow-B), ligation time point analysis was performed by varying the adapter amount. This was performed using our ssRNA-seq pipeline. *n* = 3; error bars = Standard Deviation

Our current protocol for library construction includes a bead purification step after each of the library construction reactions: end-repair, A-addition and ligation (Fig. 1). The reagents for our protocol are sourced from New England Biolabs (NEB). Recently, various commercial kits have emerged in which one or more of the bead purifications are removed and in some cases, two reactions are coupled into the same step. NEB has three different products representing these workflows (Fig. 7a). To evaluate the trade-off between library yield and streamlined workflow, these products were compared using our PCR-free protocol starting with the same sheared and size-selected HL60 gDNA. The first product employs bead clean ups after each of the library construction steps; the second removes the bead purification after A-tailing; and the third couples end-repair and A-tail reactions and enables ligation without a cleanup in between. The second product has the reaction components in one premix and, importantly, has a proprietary ligation enhancer component. This formulation gave the highest yield (Fig. 7b) with comparable sequencing data quality to the others (Additional file 5: Table S3). The yield using this chemistry was higher than that of others from different vendors that are representative of the three work flows (data not shown). The minimum input amount tested for PCR-free libraries was 250 ng using the newly optimized protocol. The robustness of the protocol has led to an ongoing assessment of even lower input amounts for the PCR-free genome pipeline. Beyond HL60, this new protocol was shown to be more robust for 3 other gDNA sources from different clinical samples (Additional file 10: Figure S6). While the current manuscript was in revision, NEB has released version 2 (NEBNext Ultra II) of their most streamlined workflow. We have not assessed this product fully even though our preliminary data suggests that library yield obtained using this protocol is comparable to the yield attained using the most optimal protocol we identified in Fig. 7 (Additional file 11: Figure S7). Of note, these comparisons did not include the respective PCR modules of the various library construction kits and instead we applied our current PCR module using Thermo Fisher’s Phusion enzyme to all protocols.

### Optimization of ligation time and adapter amount

Virtually all commercial kits for library construction employ a short ligation time (~15 min). We have observed that even with some of these kits, longer ligation times improve library yield significantly with proportional increase in library diversity (data not shown). However, ligation time could be reduced without drastic loss in library yield when a higher adapter to insert ratio is applied (Fig. 7c). Based on these data, we have chosen a 15 min optimized ligation with the NEB premix library construction chemistry to be our preferred protocol due to the improved workflow and increased library yield. These data also suggests that ligation time needs to be controlled so that ‘batch effects” due to yield (and hence library diversity) are avoided.

### Optimal mRNA isolation

The mRNA isolation technique we have been employing is the MultiMACS procedure (Miltenyi Biotec, Germany), which uses magnetic oligo-dT beads in a 96 mini-column array format [20]. We have successfully used this technique and over the years have further optimized and automated the procedure (Additional file 12: Figure S8, Additional file 13: Figure S9 and Additional file 14: Figure S10). This protocol gave high quality libraries for total RNA inputs that ranged between 500 ng and 5000 ng (Additional file 15: Figure S11). However, the major hurdle in lowering the required input further was high rRNA content. For input amounts between 25 ng to 200 ng, rRNA content varied between 10% and 60% in contrast to higher input range which ranged 0.2–10% (Additional file 5: Tables S4 and S5).

To evaluate alternative mRNA isolation methods with the aim of reducing ribosomal RNA content, we compared our latest MultiMACS-based protocol with NEB’s mRNA module and Biooscientific’s NEXTflex mRNA isolation protocol. UHR RNA input amounts tested ranged from 25 ng to 1000 ng. For all inputs tested, the two newer protocols gave much higher cDNA yield for the 1000 ng input relative to the modified MultiMACS protocol (Additional file 16: Figure S12A). Assessment of 18S rRNA to GAPDH mRNA mean expression ratio by qPCR indicated that the two protocols had much lower rRNA content with the NEB protocol providing the lowest values (Additional file 16: Figure S12B). Encouraged by these data, we made libraries representing these protocols at the various input amounts and sequenced them in a lane of HiSeq 2000. All library quality metrics favored all three protocols for input amounts 500 and 1000 ng (Additional file 5: Table S5). Consistent with the qPCR data, % ribosomal RNA was <5% for both of the new protocols with <500 ng input range whereas the multiMACS protocol gave 48–68% ribosomal for these input amounts (Additional file 5: Table S5). The NEB protocol was at 0.1–0.5% rRNA for all input amounts where as the NEXTflex protocol gave 0.2–4% rRNA for all input amounts with the higher end representing lower input libraries (Additional file 5: Table S5). Consistent with the data on cDNA yield, the libraries from the two new protocols also gave generally higher library quality in terms of transcript diversity and other quality metrics (Additional file 5: Table S5). Since the % ribosomal was consistently lower for all input amounts with the NEB protocol, this was chosen as our final mRNA isolation module.

### The new pipeline produces high quality libraries from 25 to 1000 ng Total RNA input

**Fig. 8:**
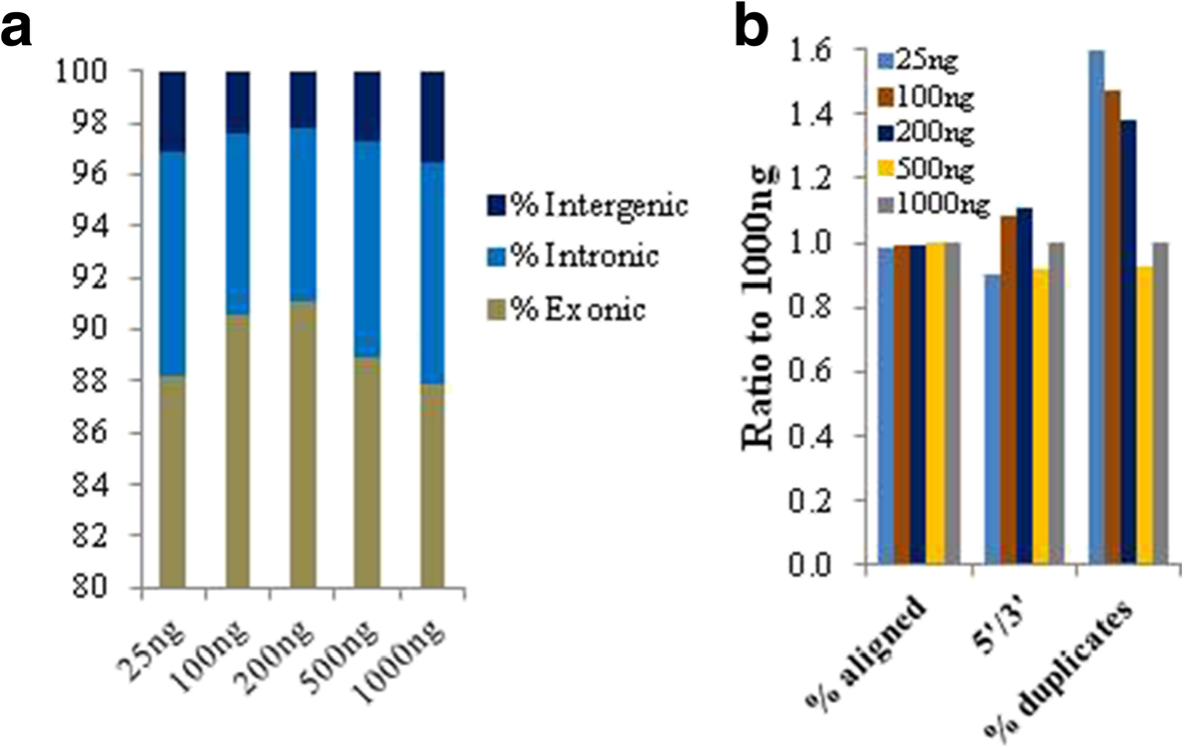
UHR total RNA input titration using the new ssRNA-seq pipeline. **a** Comparable mapping of reads to various transcriptome catagories. **b** Other alignment-based metrics. Y-axis represents the value for each of the inputs divided by that of the 1000 ng for a given metric

The aforementioned optimizations led to a new pipeline. We next assessed whether the lower input (e.g. 25 ng) libraries are indeed qualitatively comparable to the higher input (e.g. 1000 ng) libraries when using this pipeline. Relative proportions of reads that mapped to exonic, intronic and intergenic regions were comparable between the input amounts (Fig. 8a). All libraries displayed >95% alignment rates (Additional file 5: Table S5), which was comparable across the input amounts tested as low as 25 ng (Fig. 8b). All libraries had >98% strand-specificity with comparable base error rates (Additional file 5: Table S5). 3′-end bias is one caveat that is usually associated with poly (A) capture-based protocols. 5′-end to 3′-end ratios were calculated to measure the extent of this bias and were found to be within 0.87–1.1 for all the libraries (Additional file 5: Table S5). While we expect these numbers to be different for RNA samples with variable quality, these results suggest that the lowering of the input amount per se is not going to be a significant factor. The only metric where we saw a significant difference is % duplicates, which was ~1.5-fold higher for 25-200 ng input amounts as compared to 500-1000 ng input range (Fig. 8b).

One major application of RNA-seq is quantification of gene expression at the transcript level. Thus, we wanted to investigate if the low input libraries represented the relative amounts of transcripts of various expression levels rather accurately. To gauge this, we undertook two approaches. First, we calculated the correlation of expression between the different input amounts for the entire complement of the UHR transcriptome. Second, we evaluated how well the observed levels of ERCC spike-in synthetic RNAs correlated with the “ground truth” expected levels. For the latter, we spiked UHR RNA with ERCC spike-in proportional to the input amount at a constant final % value. The spiking was done just before mRNA isolation and thus the readout on the ERCC data is indicative of the aggregate of all protocols applied starting with the mRNA isolation module. Pair-wise Pearson’s correlation values indicated very high correlation of expression levels between the various input amounts (Additional file 17: Figure S13, upper panel). Average correlation relative to the 1 μg input was 0.987 for the 25 ng input, 0.989 for the 100 ng input, 0.989 for the 200 ng input and 0.992 for the 500 ng input. ERCC spike-in correlation was also in agreement with this UHR data with expression correlation of observed vs expected being 0.94–0.98 for each of the RNA input amounts (Additional file 17: Figure S13, lower panel). The correlation values within technical replicates for each input amount was 0.989–0.999 for the UHR data (Additional file 17: Figure S13, upper panel), suggesting a very high reproducibility of the new pipeline.

**Fig. 9:**
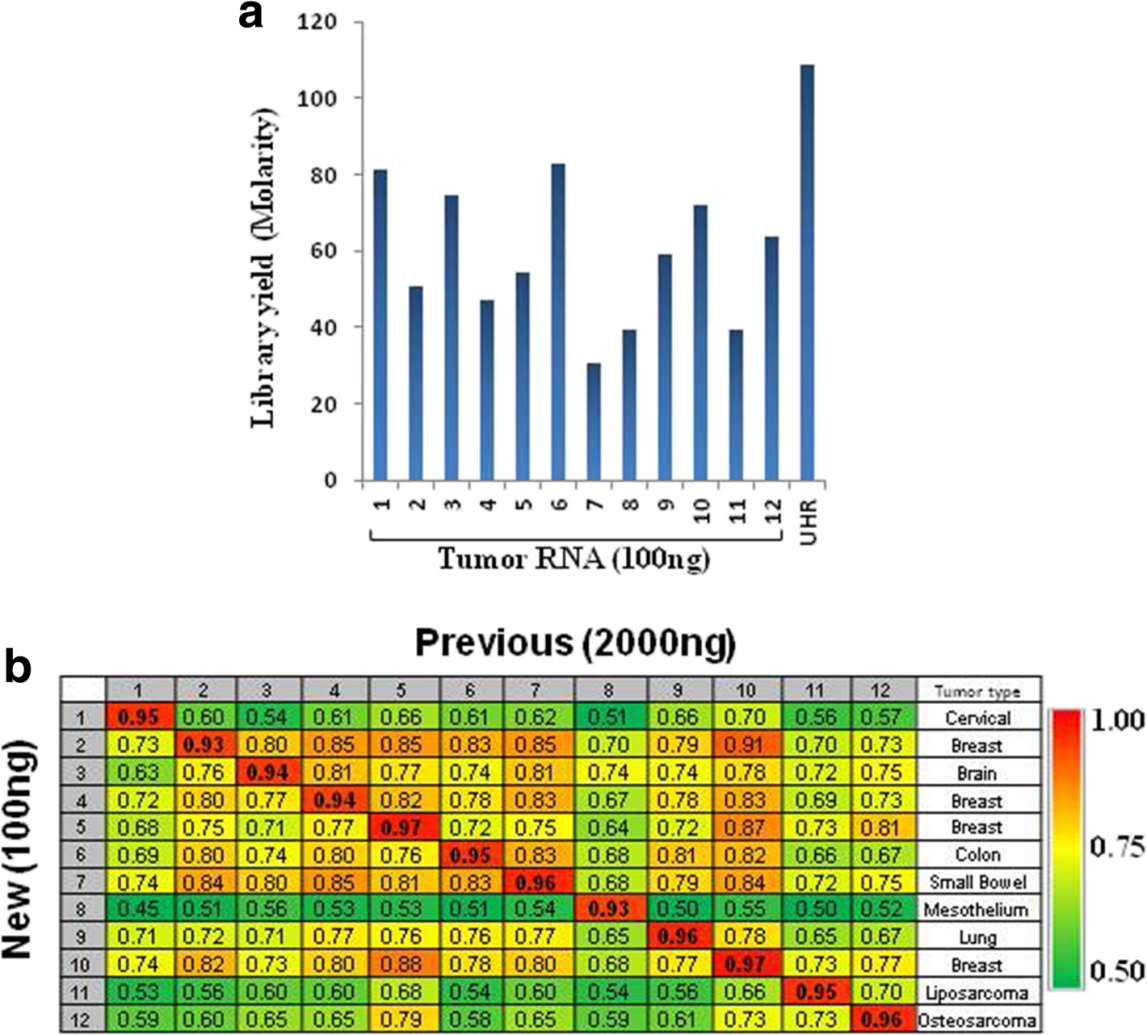
Evaluation of the new ssRNA-seq pipeline using RNA from tumor samples. 100 ng total RNA from 12 different tumor samples was used as input to generate libraries using the new protocol. Adapter-ligated libraries were enriched with 13 cycles of PCR. **a** Library yield. **b** Correlation of expression. Pearson’s correlation coefficient was calculated pair-wise showing higher correlation between the new lower input libraries and their previous higher input counterparts

We next wanted to validate the new ssRNA-seq pipeline for lower RNA input beyond UHR using RNA from tumor samples. To do this, we tapped into a set of human clinical tumour samples which were obtained as part of an ongoing personalized oncogenomics project at our centre [14]. 100 ng total RNA from twelve such samples, representing a variety of tissues, was used as a starting material. Some of the tissues were embedded in Optimal Cutting Temperature compound (OCT) while others were from fresh frozen specimens. The resulting libraries from each of the 12 samples were at an order of magnitude or higher relative to the minimum concentration required for standard Illumina sequencing (Fig. 9a). Post-alignment metrics of the sequencing data are shown in Additional file 5: Table S6. Included in these analyses is also data from libraries that were previously generated from the same samples from higher Total RNA input (2000 ng). The high input libraries were generated using the same protocols with the exception that mRNA isolation was according to the MultiMACS procedure described above. Additional file 5: Table S6 shows that the duplicate rate, % rRNA, and % intergenic is lower for the newer libraries. However, 3′-end bias appears to be slightly higher for the newer libraries with the average 5′/3 value being 0.87 versus 1.05. This is not due to input RNA quality differences as the RNA integrity was comparable (Additional file 18: Figure S14) even though degradation during subsequent DNase treatment and mRNA isolation steps cannot be ruled out. Input amount difference (100 ng versus 2000 ng) may be plausible proximal factor as we have previously observed upon UHR input titration that 5′/3′ values are variable depending on the input amount (Additional file 5: Table S5). An alternative explanation is that 5′/3′ may be confounded by non-specific capture of RNAs during mRNA isolation. Indeed, average % rRNA value and % intergenic were 9.4% and 3.8%, respectively, for the previous libraries (Additional file 5: Table S6). In contrast, the new libraries displayed 0.09%and 2.2%, respectively. Consistent with this possibility is the observation that the 5′/3′ values are reduced by a constant proportion (~ 20%) (Fig. 9b). This is despite the range of the ratios for the previous libraries being as diverse as 0.86 and 1.5 (Additional file 5: Table S6).

Expression of thousands of genes is shown to display a very high correlation between the two sets of libraries with Pearson’s correlation coefficient of 0.93–0.97 (Fig. 9b) where as expression correlation values using the same input and protocol from different tumor types were as low as 0.5. Furthermore, expression correlation between the same tumor types ranged between 0.83 and 0.91 for both input amounts/protocols. Additional support for the notion that our new protocol provides expression data that is biology-driven (as opposed to protocol/input amount- driven) comes from hierarchical clustering data that shows segregation of clades in a sample-dependent manner (Additional file 19: Figure S15).

## Conclusions

Over all, we have described here a systematic optimization and evaluation of various modules in ssRNA-seq sample preparation for Illumina sequencing that included mRNA isolation, cDNA synthesis, bead-based purifications and size selection as well as library construction chemistry. The outcome of these multifaceted improvements has enabled a streamlined, high throughput pipeline that typically yields high quality libraries at tens of nano-molar concentration with 10–13 cycles of PCR from as low as 25 ng total RNA input. Taken together, the pipeline described here will allow the rapid processing of low input samples for transcriptomic studies.

## Additional files

Additional file 1: mRNA isolation. (DOCX 75 kb)

Additional file 2: cDNA synthesis. (DOCX 159 kb)

Additional file 3: library construction. (DOCX 129 kb)

Additional file 4: Figure S1. Superscript II titration. 3.5 μg UHR was used as input Total RNA followed by multiMACS mRNA isolation. Various amounts of Superscript II were used in a 25 μL total reaction volume of 1st strand synthesis. “New” and “old” denote two different lots of the enzyme. (A) cDNA yield. (B) Duplicate rate. Strand-specific libraries were generated from the cDNA samples shown in (A). Libraries were pooled and sequenced using PE75. 20 million reads from each of the libraries were sampled for calculating duplicate rate. (JPEG 31 kb)

Additional file 5: Table S1. Alignment-based metrics for reverse transcriptase comparisons. **Table S2.** Alignment-based metrics for the size selection experiment. **Table S3.** Alignment-based metrics for the comparison of library construction kits. **Table S4.** Alignment-based metrics for the intermediate ssRNA-seq pipeline. The protocol evaluated includes all changes (1st strand cDNA synthesis, optimal bead purifications, new library construction chemistry with modified ligation condition, bead-based size selection, and UNG treatment) with the exception of the mRNA isolation improvements. **Table S5.** Alignment-based metrics for the final ssRNA-seq pipeline using UHR. **Table S6.** Alignment-based metrics for the final ssRNA-seq pipeline using tumor samples. (XLS 62 kb)

Additional file 6: Figure S2. Reverse transcriptases and UHR sequence divergence. UHR whole transcriptome data was assessed for divergence of sequence relative to a compendium of human reference comprised of known single nucleotide polymorphisms (SNPs). *n* = 3; error bars = Standard Deviation. (JPEG 24 kb)

Additional file 7: Figure S3. Larger strand-specific library sizes are generated using 1:1 post-ligation and post- ﻿UNG bead-based purifications. (A) gDNA libraries. Upper panel is a trace representing size profile of sheared HL60 gDNA (replicates are overlayed). 30 ng was used for 200 nt-gap library construction where post-ligation clean up was performed twice with 1:1 ratio of bead volume to DNA volume. Lower panel is size profile of final libraries. (B) Strand-Specific cDNA libraries. Upper panel is a trace representing size profile of sheared double-strand cDNA. cDNA was generated from 2 μg UHR t﻿otal RNA followed by multiMACS mRNA isolation. Post-ligation clean up was performed once with 1:1. Post-UNG was also once with 1:1. Lower panel is size profile of final libraries. Note that the strand-specific cDNA libraries are significantly larger than the gDNA libraries despite the sheared starting material being of similar size profile. (JPEG 50 kb)

Additional file 8: Figure S4. UNG digestion improves library yield. Libraries were made from the indicated UHR input amounts in the presence or absence of UNG in the PCR reaction. UNG from two different vendors was used. *n* = 2. (JPEG 26 kb)

Additional file 9: Figure S5. The performance of our tool for calculating strand-specificity. (JPEG 23 kb)

Additional file 10: Figure S6. Library construction chemistry comparison using tumor samples. Two NEB workflows were evaluated. The first has bead clean up after each of the library construction steps (A); the second has the bead cleanup after A-tail removed (B). Input gDNA was from three different clinical samples (Source 1–3). (JPEG 27 kb)

Additional file 11: Figure S7. Library yield is improved with the latest streamlined NEB library construction protocol (NEBUltraII). 100 ng HeLa gDNA was used as input and 6 cycles of PCR was applied. PCR module with Phusion enzyme was the same for both. NEB-B is the most optimal protocol identified as shown in Fig. 7. (JPEG 20 kb)

Additional file 12: Figure S8. Oligo-dT bead titration for MultiMACS mRNA isolation and its effect on % rRNA and mRNA/cDNA yield. (A) % rRNA as assessed by qPCR. Oligo-dT bead amount was titrated. Indicated amounts are expressed relative to manufacturer specified amount (1×). qPCR was applied to measure levels of 18S rRNA and GAPDH mRNA. The ratio of 18S to GAPDH levels was calculated based on the Pfaffl method (Pfaffl MW, 2001) and is shown graphically. (B) Duplicate rate upon sequencing. For selected bead amounts, libraries were generated and sequenced. Y-axis is ratio of duplicate rate calculated relative to the manufacturer specified amount (1×). (C). Oligo-dT bead amount and cDNA yield. Of note, this represents the latest lots of the beads which appear to have significantly underperformed where the effect of lower bead amount is most drastic. (JPEG 56 kb)

Additional file 13: Figure S9. Workflow and automation of various MiltiMACS mRNA isolation formats and on-column cDNA synthesis. Streamlined MultiMACS that does not require precipitation before cDNA synthesis (no ppt) versus another version that requires precipitation (ppt) as well as on-column cDNA synthesis are depicted in (A). In (B), the integration of the MutiMACS unit with Hamilton Nimbus Microlab liquid handler is shown. (JPEG 66 kb)

Additional file 14: Figure S10. Comparison of various MiltiMACS mRNA isolation formats and on-column cDNA synthesis. Streamlined MultiMACS that does not require precipitation before cDNA synthesis (no ppt) versus another version that requires precipitation (ppt) as well as on-column cDNA synthesis were compared. For the latter, two different cDNA synthesis mixes were used: one that comes with the Miltenyi kit that involves dT-priming and another that is based on our protocol which is spiked with random hexameres (in-house). (A) Detection of gene expression. Number of genes whose expression is detected at various degrees of coverage is shown for all the four conditions. (B) 3′-end bias. X-axis values represent proportion of genes whose coverage shows a certain degree of asymmetry. When the proportion with asymmetric coverage (x) is 0, there is no bias at all. When x > 0, there more bias towards the 3′-end and vice versa. (C) Mapping to various transcriptomic regions. *n* = 3. (JPEG 49 kb)

Additional file 15: Figure S11. Total RNA input titration using the intermediate ssRNA-seq pipeline**.** The protocol evaluated includes all changes (1st strand cDNA synthesis, optimal bead purifications, new library construction chemistry with modified ligation condition, bead-based size selection, and UNG treatment) with the exception of the mRNA isolation improvements. (A) Comparable mapping of reads to the human genome. (B) Comparable mapping of reads to various transcriptome catagories. (C) High correlation of expression between lower input and higher input libraries. Pearson’s correlation coefficient was calculated pair-wise as indicated. Heat map was generated for the resulting values with color intensity representing the degree of correlation as per the depicted legend. *n* = 3 for all inputs except for 2μg and 5μg input amounts where only duplicates were represented. Error bars = Standard Deviation. (JPEG 27 kb)

Additional file 16: Figure S12. Comparison of mRNA isolation kits. The modified MultiMACS protocol with 1/16th bead amount was compared to two other kits (NEXTflex and NEB). Various UHR inputs were used. (A) cDNA yield. (B) % rRNA as assessed by qPCR. Details are as in Additional file 12: Figure S8. All three are shown in the left panel. Right panel shows just the NEXTflex and NEB on a different scale. (JPEG 37 kb)

Additional file 17: Figure S13. High correlation of expression between lower input and higher input libraries. Upper panel is for UHR data. Lower panel: High correlation of observed versus expected levels of ERCC spike-in synthetic RNAs. Pearson’s correlation coefficient was calculated pair-wise among the libraries (UHR data) and between what was measured in the libraries versus theoretically expected levels for the ERCC RNAs. *n* = 2. (JPEG 50 kb)

Additional file 18: Figure S14. RNA integrity comparisons between the input RNAs for the previous and newer libraries. RNA integrity (RIN) is based on Agilent RNA Nano assay. (JPEG 25 kb)

Additional file 19: Figure S15. Hierarchical clustering data that shows segregation of clades in a sample dependent manner as opposed to segregation by input amount RNA. Samples are as in Fig. 9. LI = low input; HI = high input. (JPEG 50 kb)

## Abbreviations

ERCC: External RNA Controls Consortium
mRNA: Messenger RNA
NGS: Next Generation Sequencing
RNA-Seq: RNA sequencing
rRNA: Ribosomal RNA
RT: Reverse Transcriptase
SSII: Superscript II
ssRNA-seq: Strand-specific RNA-seq
