# Supplementary material for: Increasing quality, throughput and speed of sample preparation for strand-specific messenger RNA sequencing
Source: BMC Genomics. 2017 Jul 5;18:515. doi: 10.1186/s12864-017-3900-6 (PMC5499059; doi:10.1186/s12864-017-3900-6)
Supplement: Supplementary file 1 — mRNA isolation. (DOCX 75 kb) [file 12864_2017_3900_MOESM1_ESM.docx]

Magnetic bead-based mRNA isolation

EP 2)

# Purpose

To capture poly (A) mRNA from Total RNA using mRNA isolation kit from New England Biolabs (NEB). This process represents mRNA isolation V2.0 and replaces the process of MultiMACs RNA isolation described in LIBPR.0057 and LIBPR.0118.

# Scope

All procedures are applicable to the BCGSC Library Core and the Library TechD groups.

# Policy

This procedure will be controlled under the policies of the Genome Sciences Centre, as outlined in the Genome Sciences Centre High Throughput Production Quality Manual (QM.0001). Do not copy or alter this document. To obtain a copy see a QA associate.

# Responsibility

It is the responsibility of all personnel performing this procedure to follow the current protocol. It is the responsibility of the Group Leader to ensure personnel are trained in all aspects of this protocol. It is the responsibility of Quality Assurance Management to audit this procedure for compliance and maintain control of this procedure.

# References

| Document Title | Document Number |
| --- | --- |
| NEBNext Poly(A) mRNA Magnetic Isolation Module | E7490L |

# Related Documents

| Document Title | Document Number |
| --- | --- |
| Operation and Maintenance of the Agilent 2100 Bioanalyzer for DNA samples  Operation and Maintenance of the Agilent 2100 Bioanalyzer for RNA Samples  Operation and Maintenance of the LabChipGX for RNA samples using the HT RNA Assay  Total RNA Normalization on Hamilton Nimbus | LIBPR.0017  LIBPR.0018  LIBPR.0052  LIBPR.0121 |

# Safety

All Laboratory Safety procedures will be complied with during this procedure. The required personal protective equipment includes a laboratory coat and gloves. See the material safety data sheets (MSDS) for additional information.

# Materials and Equipment

| Name | Supplier | Number |
| --- | --- | --- |
| Fisherbrand Textured Nitrile gloves - large | Fisher | 270-058-53 |
| RNAse Zap | Ambion | 9780 |
| Ice bucket – Green | Fisher | 11-676-36 |
| Wet ice | In house | N/A |
| RNAse free 1.5 mL eppendorf tube | Ambion | 12400 |
| Gilson P2 pipetman | Mandel | GF-44801 |
| Gilson P10 pipetman | Mandel | GF-44802 |
| Gilson P20 pipetman | Mandel | GF23600 |
| Gilson P200 pipetman | Mandel | GF-23601 |
| Gilson P1000 pipetman | Mandel | GF-23602 |
| Mandel P200 DF200 tips | Mandel | GF-F171503 |
| Mandel P1000 DF1000 tips | Mandel | GF-F171703 |
| VX-100 Vortex Mixer | Rose Scientific | S-0100 |
| 200µL Rainin tips | Rainin | RT-L200F |
| 20µL Rainin tips | Rainin | RT-L10F |
| 200µL Pipet-Lite | Rainin | L12-200 |
| 20µL Pipet-Lite | Rainin | L12-20 |
| 1250 µL pipette tip, 96tips/rack, 480 tips/cs filter sterile | Mandel Scientific | TM-4445 |
| Pipette-VIAFLO 8 Channel | Mandel Scientific | TM-4124 |
| Large Kimwipes | Fisher | 06-666-117 |
| Black ink permanent marker pen | VWR | 52877-310 |
| Bench Coat (Bench Protection Paper) | Fisher | 12-007-186 |
| Small Autoclave waste bags 10”X15” | Fisher | 01-826-4 |
| DNAse1 Amplification Grade 100U | Invitrogen | 18068-015 |
| DEPC water | Ambion | 9922 |
| Mini-centrifuge | Eppendorf | 5417R |
| Thermo Scientific 0.2mL Ultra Rigid Skirted 96-well PCR Plate | Thermoscientific | AB1000-150s custom |
| Deep-well, 96-well, 1.2 mL, U bottom, low pro, 50/cs | Fisher Scientific | AB1127 |
| **NEBNext Poly(A) mRNA Magnetic Isolation Module** | **New England Biolabs** | **E7490L** |
| RNA MagClean DX | ALINE Biosciences | C-1005 |
| Sterile Filtered Conductive 50µL Tips in Frames | Hamilton | 235979 |
| Sterile Filtered Conductive 300µL Tips in Frames | Hamilton | 235938 |
| Tape Pads | Qiagen | 19570 |
| Foil Tape | VWR | 60941-126 |

1. **GENERAL GUIDELINES**
2. **General guidelines and input material**
   1. The recommended input material for this procedure is 100-1000ng Total RNA. Input volume to be requested from collaborators should be a maximum of 20µL to have suitable concentration for QC. The actual input volume for the first reaction is 35µL/well in DEPC H_2_0 in a 96-well plate.
   2. The positive control for this procedure is 500 ng Universal Human Reference RNA (UHR or FG031). Please also add a positive control (UHR) that is the same amount as the rest of the normalized plate. The negative control is DEPC H_2_O.
   3. The beads (**RNA MagClean DX**) to be used in this protocol are different from those used for other protocols as they are certified to be RNase-free.
   4. Ensure proper personal protective equipment is used when handling sample plates, reagents and equipment. Treat everything with, and adhere to, strict RNA handling techniques.
   5. Wipe down the assigned workstation, pipetman, tip boxes and small equipment with RNAse Zap (Ambion) followed by DEPC-treated water. Ensure you have a clean working surface before you start.
   6. Double check the QA release and/or expiry date of each reagent and enzyme.
   7. Reactions in plates should never be vortexed and plate covers are never to be re-used.
   8. Retrieve and thaw all reagents at room temperature. Once thawed, pulse-vortex, quick spin and keep reagents on ice. Enzymes should be left in the freezer until ready to use.
   9. Ensure the waste bag for the Nimbus is empty.
   10. **Brews are prepared and dispensed manually**. Note that what is dispensed into the final brew plate is the actual volume of the specified volume for each step below without any dead volume.
   11. Aline RNA MagClean DX Beads are manually dispensed into wells in a 1.2 mL plate (AB1127). 20 µL dead volume should be factored in. Ensure that you include 25 mL dead volume for 70% ethanol and DEPC H_2_O on top of what is required for the actual washes and elution, respectively.
   12. The Nimbus adds sample in a given reaction to the brew and bead cleanups are performed on Nimbus. Follow the prompts and lay out from the Nimbus programs to execute a particular step.
   13. The Nimbus mixes at 80% of total volume 10 times.
   14. Note that where it is specified that you proceed immediately to the next step, plates can be briefly placed on ice (not more than 30min) in the case of emergency. Make an active attempt to proceed as immediate as possible.
3. **PROCEDURE**

**1. Upstream Preparation**

- 1. Retrieve the plate containing Total RNA. If stored in -80°C, thaw it on ice followed by a quick spin at 4°C, 700g for 1min. Place it on ice.

1. **Input QC:**
   1. For total nucleic acid as input, it is recommended that contaminating gDNA is first quantified using Qubit (LIBPR.0030) or Quant-IT (LIBPR.0108). RNA QC can be skipped at this stage (200-400ng gDNA should generally give more than 200ng RNA after DNase treatment). The RNA/DNA mixture entering this DNase treatment should contain <400ng gDNA. Normalization to 200-400ng gDNA should be performed using Nimbus (LIBPR.0121) or manually if deemed appropriate.
   2. For purified RNA as input, RNA is quantified using Agilent RNA Nano (LIBPR.0018) or Caliper RNA (LIBPR.0052) assays. For Caliper QC, 2 µL of sample is used instead of the 1 µL specified in LIBPR.0052. An additional modification of the Caliper QC is the RNA is diluted in 18 µL buffer after denaturation (instead of 46 µL specified in the standard protocol). Also, select “AB1000_2.5mm” as the total volume is lower for the Caliper plate. The resulting concentrations are considered as they are without any dilution factor. Log into Nimbus Program as follows to transfer 2 µL of the RNA for Caliper QC:

| C:\Program Files (x86) > Hamilton Company > Methods > Production > mRNA Isolation v2.0 > **mRNA Isolation v2.0 –** ***Scheduler.wfl** > RUN **> Caliper Transfer** |
| --- |

“*” Every time you see “**Scheduler.wfl”** addition to the name of the program, it means that you will have to select “work flow” option from the drop down menu at the bottom right corner of the window.

Normalize samples from purified RNA to the same RNA amount within 100-1000ng using Nimbus (LIBPR.0121) or manually if deemed appropriate.

LIBPR.0121 Total RNA Normalization on the Hamilton Nimbus

1. **1^st^ DNase I Treatment: Remove contaminating gDNA in samples**
   1. All samples should be DNase treated as follows (regardless of whether or not they were treated by collaborators). Samples should be in 35µL.
      1. For purified RNA that was extracted using protocols such as Qiagen’s where RNA is separated from gDNA, the reaction set up for 1 reaction is shown below. Use LIMS to generate the brew calculator for this step. LIMS Calculator (intact RNA): RBD_1st DNase_1U

| **Solution** | **µL (per 1rxn)** |
| --- | --- |
| RNA | 35 |
|  |  |
| DEPC H_2_0 | 9  1^st^ DNase Mix  (15 µL) |
| 10X DNase Buffer | 5 |
| DNase I Enzyme | 1 |
|  |  |
| **Total Reaction volume** | **50** |

- - 1. For Total Nucleic acid, the reaction set up for 1 reaction is shown below. LIMS Calculator: RBD_1st DNase_5U

| **Solution** | **µL (per 1rxn)** |
| --- | --- |
| RNA | 35 |
|  |  |
| DEPC H_2_0 | 5  1^st^ DNase Mix  (15 µL) |
| 10X DNase Buffer | 5 |
| DNase I Enzyme | 5 |
|  |  |
| **Total Reaction volume** | **50** |

- 1. Make sure to mix each reagent well and quick spin before adding to the brew. Add enzyme last.
  2. Prepare the reaction brew in a non-stick tube and check off reagents as they are added. Mix the brew by repeated pulse-vortexing followed by a quick spin.
  3. Log into Nimbus Program as follows to add the RNA to 15 µL of the DNase mix:

| C:\Program Files (x86) > Hamilton Company > Methods > Production > mRNA Isolation v2.0 > **mRNA Isolation v2.0 – Scheduler.wfl** > RUN **> DNAse** |
| --- |

- 1. After Nimbus program completion, seal the plates and quick spin at 4ºC for 1 minute. Inspect the reaction plates for any variations in volume.
  2. Incubate for 15 min at room temperature. Note: *This is NOT a safe stopping point; proceed to the next step immediately.*
  3. Log into Nimbus Program as follows to add the RNA to 5 µL of 25mM EDTA:

| C:\Program Files (x86) > Hamilton Company > Methods > Production > mRNA Isolation v2.0 > **mRNA Isolation v2.0 – Scheduler.wfl** > RUN **> EDTA** |
| --- |

- 1. After Nimbus program completion, seal the plates and quick spin at 4ºC for 1 minute. Inspect the reaction plates for any variations in volume.
  2. In the Tetrad thermocycler, incubate the plate at 65˚C for 10 min. During incubation, dispense 110 µL of Beads per well (1.2 mL plate) in preparation for the subsequent cleanup.

TETRAD: MRNA > STOP_DN

- 1. After incubation, remove the plate and spin it at 4°C, 700g for 1min. Note: *This is NOT a safe stopping point; proceed to the next step immediately.*

1. **Post–DNase I treatment Bead clean up (use RNA MagClean DX beads)**
   1. The input volume for this step is 55 µL per well.
   2. The Nimbus will perform the cleanup of the DNase reaction using beads as follows:

| **Reaction** | ***Bead Vol manually dispensed (µL)** | **Bead Vol added by Nimbus (µL)** | **Bead Binding Time (mins)** | **Magnet Clearing Time (mins)** | **2X 70% EtOH* Wash Vol (µL)** | **Magnet Airdry Time (mins)** | **Elution Vol (µL)** | **Elution time (mins)** | **Magnet Elution time (mins)** | **Transfer Vol (µL)** |
| --- | --- | --- | --- | --- | --- | --- | --- | --- | --- | --- |
| **DNase Reaction** | **120** | **99** | **15** | **7** | **180** | **5** | **52** | **3** | **2** | **52** |

*Must be at Room Temp for a minimum of 30mins before usage; failure to do so would result in a decrease in yield

- 1. Log into Nimbus Program as follows:

| C:\Program Files (x86) > Hamilton Company > Methods > Production > mRNA Isolation v2.0 > **mRNA Isolation v2.0 – Scheduler.wfl** > RUN **> Bead Cleanup1.1 > mRNA DNase** |
| --- |

*This is a safe stopping point. If needed, the plate containing the bead cleaned RNA can be stored at* ***-80°C.***

- 1. **For Total Nucleic Acid only**, DNase-treated RNA should be quantified using Agilent RNA Nano (LIBPR.0018) or Caliper RNA (LIBPR.0052). Skip this QC for purified RNA input. For Caliper QC, 2 µL of sample is used instead of the 1 µL specified in LIBPR.0052. An additional modification of the Caliper QC is the RNA is diluted in 18 µL buffer after denaturation (instead of 46 µL specified in the standard protocol). Also, select “AB1000_2.5mm” as the total volume is lower for the Caliper plate. The resulting concentrations are considered as they are without any dilution factor. Log into Nimbus Program as follows to transfer 2 µL of the RNA for Caliper QC:

| C:\Program Files (x86) > Hamilton Company > Methods > Production > mRNA Isolation v2.0 > **mRNA Isolation v2.0 – Scheduler.wfl** > RUN **> Caliper Transfer** |
| --- |

- 1. DNase-treated RNA from Total nucleic Acid should be normalized to 100-1000ng at this stage using Nimbus according to LIBPR.0121.

1. **mRNA capture**; **manual preparation**
   1. RNA volume is 50 μL.
   2. Aliquot 15 μL of NEBNext Oligo d(T)_25_ beads into 96-well plate (ABGENE). Label the plate as “**Beads**”.
   3. Aliquot 350 μL of RNA Binding Buffer into Plate, 96-well v bottom plate (P-96-450V-C). Label the plate as “**Binding Buffer**”.
   4. Aliquot 900 μL of Wash buffer into Deep-well, 96-well, 1.2 mL, U bottom, low profile (AB1127). Label the plate as “**Wash Buffer**”.
   5. Aliquot 90 μL of Tris Buffer into 96-well plate (ABGENE). Label the plate as “**Tris Buffer**”.
2. **mRNA capture**; **on nimbus**
   1. Log into Nimbus Program as follows and follow the prompts to perform the mRNA isolation steps including incubation steps:

| C:\Program Files (x86) > Hamilton Company > Methods > Production > mRNA Isolation v2.0 > **mRNA Isolation v2.0 – Scheduler.wfl** > RUN **> mRNA Capture** |
| --- |

The detailed steps, which the Nimbus will be performing within the program selected as above are described in Appendix A.

1. **The mRNA is now ready for cDNA synthesis.**

**Appendix A: Detailed steps performed by Nimbus within the program “mRNA Capture”**

1. Add100 μL of RNA Binding Buffer from the “**Binding Buffer**” plate to the “**Beads**” plate. Mix (80% Total volume=92 μL) 10 times.
2. Place the beads plate on magnet for 2 minutes.
3. Remove the supernatant.
4. Remove the plate from the magnet.
5. Repeat steps 1–4.
6. Add 50 μL of RNA Binding Buffer from the “**Binding Buffer**” plate.
7. Add 50 μL of **total RNA sample** using same tips. Mix (80% Total volume=80 μL) 10 times.
8. *Place the plate on a thermal cycler (tetrad: MRNA>DENATUR) and incubate 65°C for 5 minutes and hold at 4°C to denature the RNA and facilitate binding of the poly-A mRNA to the beads.*
9. *Remove the plate when the temperature reaches 4°C (2min).*
10. Mix (80% Total volume=80 μL) 10 times.
11. Incubate at room temperature for 5 minutes to allow the mRNA to bind to the beads.
12. Place the plate on the magnet at for 2 minutes.
13. Remove the supernatant.
14. Remove the plate from the magnet.
15. Wash the beads by adding 180 μL of Wash Buffer from the “**Wash Buffer**” plate. Mix (80% Total volume=160 μL) 10 times.
16. Place the plate on the magnet for 2 minutes.
17. Remove the supernatant.
18. Remove the plate from the magnet.
19. Repeat steps 15–18.
20. Add 50 μL of Tris Buffer from the “**Tris Buffer**” plate. Mix (80% Total volume=40 μl) 10 times.
21. *Place the plate on the thermal cycler (tetrad: MRNA>ELUTE1) and incubate at 80°C for 2 minutes, then hold at 25°C to elute the Poly-A mRNA from the beads.*
22. *Remove the plate from the thermal cycler when the temperature reaches 25°C (2minutes).*
23. Add 50 μL of RNA Binding Buffer from the “**Binding Buffer**” plate to allow the mRNA to re-bind to the beads. (80% Total volume=80 μL) 10 times.
24. Incubate the plate at room temperature for 5 minutes.
25. Place the tube on the magnetic rack at room temperature for 2 minutes.
26. Remove the supernatant.
27. Remove the plate from the magnet.
28. Wash the beads by adding 180 μL of Wash Buffer from the “**Wash Buffer**” plate. Mix (80% Total volume=160 μL) 10 times.
29. Place the plate on the magnet for 2 minutes.
30. Remove the supernatant.
31. Remove the plate from the magnet.
32. Repeat steps 28–31.
33. Elute mRNA from the beads by adding 36 μL of the Tris Buffer from the the “**Tris Buffer**” plate. Mix (80% Total volume=29 μL) 10 times.
34. *Incubate the plate in a thermocycler (tetrad: MRNA>ELUTE2) at 80°C for 2 minutes.*
35. Place the plate on magnet.
36. Transfer the supernatant (36 μL) into a clean 96-well plate (ABGENE) (“**mRNA plate**”)

**Appendix B: LIMS SOP**

1. Start of Plate Library Construction
2. Bioanalyzer Run / Caliper Run. QC Category: Total RNA QC
3. Mag Bead Based mRNA Isolation . RNA_strategy is “strand specific” and the pipeline is Strand Specific Transcriptome 3.0: SSTRA_3.0
