# Supplementary material for: Increasing quality, throughput and speed of sample preparation for strand-specific messenger RNA sequencing
Source: BMC Genomics. 2017 Jul 5;18:515. doi: 10.1186/s12864-017-3900-6 (PMC5499059; doi:10.1186/s12864-017-3900-6)
Supplement: Supplementary file 2 — cDNA synthesis. (DOCX 159 kb) [file 12864_2017_3900_MOESM2_ESM.docx]

Manual Plate-based Strand-specific cDNA Synthesis using Maxima H Minus

EP 2)

# Purpose

To synthesize cDNA for strand specific RNA-Seq (WTSS) Illumina library construction using Maxima H Minus Reverse Transcriptase from Thermo-Fisher.

# Scope

All procedures are applicable to the BCGSC Library Core group and the Library TechD group.

# Policy

This procedure will be controlled under the policies of the Genome Sciences Centre, as outlined in the Genome Sciences Centre High Throughput Production Quality Manual (QM.0001). Do not copy or alter this document. To obtain a copy see a QA associate.

# Responsibility

It is the responsibility of all personnel performing this procedure to follow the current protocol. It is the responsibility of the Group Leader to ensure personnel are trained in all aspects of this protocol. It is the responsibility of Quality Assurance Management to audit this procedure for compliance and maintain control of this procedure.

# References

| Reference Title | Reference Number |
| --- | --- |
| N/A | N/A |

# Related Documents

| Document Title | Document Number |
| --- | --- |
| Operation and Maintenance of the Agilent 2100 Bioanalyzer for DNA samples | LIBPR.0017 |
| Manual Bead Clean using Ampure XP Beads | LIBPR.0073 |

# Safety

All Laboratory Safety procedures will be complied with during this procedure. The required personal protective equipment includes a laboratory coat and gloves. See the material safety data sheets (MSDS) for additional information.

# Materials and Equipment

| **Name** | **Supplier** | **Cat. No.** |
| --- | --- | --- |
| Fisherbrand Textured Nitrile gloves | Fisher |  |
| RNAse Zap | Ambion | 9780 |
| Ice bucket – Green | Fisher | 11-676-36 |
| wet ice | In house | N/A |
| RNAse free 1.5 mL eppendorf tube | Ambion | 12400 |
| Axygen PLATE PCR 96 FULLSKT | Fisher Scientific | PCR-96-FS-C |
| Axygen plate PCR 96 fullskt BRC 50/CS | Fisher | 14222327 |
| Gilson P2 pipetman | Mandel | GF-44801 |
| Gilson P10 pipetman | Mandel | GF-44802 |
| Gilson P20 pipetman | Mandel | GF23600 |
| Gilson P200 pipetman | Mandel | GF-23601 |
| Gilson P1000 pipetman | Mandel | GF-23602 |
| Diamond Filter tips DFL10 | Mandel Scientific | GF-F171203 |
| Diamond Filter tips DF30 | Mandel Scientific | GF-F171303 |
| Diamond Filter tips DF200 | Mandel Scientific | GF-F171503 |
| Diamond Filter tips DF1000 | Mandel Scientific | GF-F171703 |
| P2-20 Rainin Lite Manual 12-channel | Rainin | L12-20 |
| P20-200 Rainin Lite Manual 12-channel | Rainin | L12-200 |
| P200 Barrier Rainin tips | Rainin | RT-L200F |
| P20 Barrier Rainin tips | Rainin | RT-L10F |
| VX-100 Vortex Mixer | Rose Scientific | S-0100 |
| Large Kimwipes | Fisher | 06-666-117 |
| Black ink permanent marker pen | VWR | 52877-310 |
| Bench Coat | Fisher | 12-007-186 |
| Small Autoclave waste bags 10”X15” | Fisher | 01-826-4 |
| 70% Ethanol | In house |  |
| Mini-centrifuge | Eppendorf | 5417R |
| Peltier Thermal Cycler | MJ Research | PTC-225 |
| Plastic seal (3M) | Qiagen | 19570 |
| Aluminum Foil seal | VWR | 60941-126 |
| Maxima H Minus First Strand cDNA Synthesis Kit | Thermo-Fisher | K165B001 |
| Actinomycin D 5mg | MJS Biolynx | ENZGR3000005 |
| Actinomycin D 10 mg/mL | In house |  |
| Custom Second strand kit (5 kits) | Invitrogen | A26942 |
| [dNTP Blend, 12.5 mM with dUTP](https://products.appliedbiosystems.com:443/ab/en/US/adirect/ab;jsessionid=zGfvNZ7MqppR0Dd3mXjcPbYFWcTm9nsQ59dp133T8zQ7t7RcTk14%21-2066002630?cmd=catProductDetail&productID=N8080270&catID=601067&backButton=true), 1mL | GeneAmp | N8080270 |
| E.Coli DNA Ligase (10U/µL) | Invitrogen | 18052-019 |
| E.Coli DNA Polymerase (10U/µL) | Invitrogen | 18010-025 |
| E.Coli RNAseH (2U/µL) | Invitrogen | 18021-071 |

1. **GENERAL GUIDELINES**
2. **General guidelines and input material**

1.1 The input material for this procedure is polyA+ RNA or Ribo-depleted RNAwhich has been DNAse I treated. The input volume for this protocol is 34.3µL/well in elution buffer from Multimacs in a 96well Axygen-PCR-96-FS.

- 1. The same positive control (such as Universal Human Reference RNA) and negative controls used in the polyA+ isolation protocol or Ribo-depleted RNA should be carried into this protocol
  2. Ensure proper personal protective equipment is used when handling sample plates, reagents and equipment. Treat everything with, and adhere to, strict RNA handling techniques.
  3. Wipe down the assigned workstation, pipetman, tip boxes and small equipment with RNAse Zap (Ambion) followed by DEPC-treated water. Ensure you have a clean working surface before you start.
  4. Double check the QA release and/or expiry date of each reagent and enzyme.
  5. The 10µg/µL stock of Actinomycin D should be thawed just before 1^st^ strand synthesis brew set up. All thawed but unused Actinomycin D should be discarded to the appropriate waste container (Sybr-green waste). As well, discard any related items such as tips, tubes, etc with traces of Actinomycin D into the Sybr-green waste container.
  6. Reactions in plates should never be vortexed and plate covers are never to be re-used. Single/Multi-Channel pipettors should be used for mixing reactions in a circular mixing motion by aspirating volume from the bottom of the well and dispensing higher up at the liquid level.

1. **PROCEDURE**

**Note: ALINE beads can be used as a direct replacement of Ampure XP beads in steps that specify the use of Ampure XP magnetic beads.**

**1. First strand cDNA: Upstream Preparation**

- 1. Retrieve and thaw Thermo-Fisher reagents at room temperature: 5X First Strand (RT) Buffer, 10mM dNTP mix, Random primers ( 200ng/µL). Once thawed, pulse-vortex, quick spin and keep reagents on ice. The buffer contains DTT, make sure it is vortexed well and that all precipitate goes in to solution. Enzymes should be left in the freezer until ready to use.
  2. Retrieve and thaw 10µg/µL Actinomycin D solution at room temperature (See Appendix B). Once thawed, vortex very well and quick spin.
  3. Retrieve the plate containing polyA+ or Ribo-depleted RNA fraction. If stored in

-80°C, thaw it on ice followed by a quick spin at 4°C, 2000g for 1min. Place it on ice.

- 1. Generate the “**SS_cDNA_1st _strand_Maxima H Minus**” brew using LIMS

LIMS: Prepare Standard Solutions > SS_cDNA_1st_strand_Maxima_H_Minus > follow the prompts > Save Standard Solution

- 1. Retrieve 1D Large label from 5^th^ floor printer outside the RNA room and brew mix check-list label from RNA Room printer.
  2. If LIMS is down, enter the number of samples to be processed and print the ss-cDNA Worksheet located in:

| R:\Lib core\Work Sheets and Calculators\Strand Specific\Manual\Manual Plate-based Strand-specific cDNA synthesis with Maxima H Minus |
| --- |

1. **Heat Denaturation of mRNA**
   1. Heat denature the poly (A) + or Ribo-depleted RNA at 70°C for 5 min using the tetrad thermo-cycler.

- 1. After 5 min of denaturation, chill the plate on ice for at least 1min. Spin down the plate at 4°C, 2000g for 1min and place it back on ice.

1. **First strand cDNA: Reaction Brew**
   1. The reaction set up for 1 reaction is shown below.

| **Solution** | **µL (per 1rxn)** |
| --- | --- |
|  |  |
| mRNA | 34.3 |
|  |  |
| 5X RT Buffer | 10  First Strand Brew Mix (15.7µL) |
| Actinomycin D (10µg/µL) | 0.2 |
| 10mM dNTP mix | 2.5 |
| Random primers (200ng/µL) | 2 |
| Maxima H Minus enzyme mix | 1 |
|  |  |
| **Total Reaction volume** | **50** |

- 1. Make sure to mix each reagent well and quick spin before adding to the brew. Add enzyme last.
  2. Prepare the reaction brew in a non-stick tube and check off reagents as they are added on the worksheet. Mix the brew by repeated pulse-vortexing followed by a quick spin. Label the First Strand brew as FS brew and place on ice until ready to use.
  3. Add 15.7µL of the FS brew into each well of a new plate.
  4. Transfer the denatured mRNA into the corresponding wells of the plate containing the 1^st^ strand reaction brew, mix 10 times, seal plate with VWR foil seal and spin down at 4°C, 2000g for 1min.
  5. In the Tetrad thermo-cycler incubate the 1^st^ strand cDNA synthesis reaction at 25°C for 10min followed by 50°C for 1h.

TETRAD: SDWTSS folder > CDNA1MAX

***Important: during incubation, continue onto section 4***

1. **Post-First Strand cDNA: Preparation**

4.1 During the 1^st^ strand cDNA synthesis reaction, retrieve the following:

- - 1. Aliquot of Ampure XP beads, 200µL/sample + dead volume. Leave at room temperature for at least 15 minutes before usage.
    2. Aliquot of 70% Ethanol (prepared using DEPC-treated water), 600µL/sample + dead volume. Leave at room temperature for at least 15 minutes before usage.
    3. 5X Second Strand buffer and 12.5mM GeneAmp dNTPs with dUTP mix. Thaw them at RT and once thawed immediately transfer to ice. Leave enzymes in the freezer until ready to use.
  1. After the 1h incubation is over, remove the plate from the thermo-cycler, spin it down at 2000g, for 1min.

1. **Post–First Strand cDNA: Ampure XP bead clean up of RNA/DNA hybrid.**
   1. The input volume for this step is 50µL per well.
   2. To clean up the 1^st^ strand cDNA synthesis reaction using Ampure XP beads, follow the modifications below and refer to:

LIBPR.0073- Manual Bead Clean using Ampure XP Beads

| **Reaction** | **Ampure XP bead* Vol (µL)** | **Bead Binding Time (mins)** | **Magnet Clearing Time (mins)** | **2X 70% EtOH* Wash Vol (µL)** | **Magnet Airdry Time (mins)** | **Elution Vol (µL)** | **Elution time (mins)** | **Magnet Elution time (mins)** | **Transfer Vol (µL)** |
| --- | --- | --- | --- | --- | --- | --- | --- | --- | --- |
| **1^st^ strand**  **ss-cDNA** | **100** | **15** | **7** | **150** | **5** | **36** | **3** | **2** | **35**** |

*must be

*Must be at Room Temp for a minimum of 30mins before usage; failure to do so would result in a decrease in yield

** The elution and transfer is set for 36µL to ensure that we’ll have sufficient and consistent resulting volume of at least 35µL in the second strand cDNA synthesis reaction.

- - 1. During the bead clean incubation steps, it is recommended to prepare the Second Strand brew (section 6).
    2. After the Magnet Elution time, transfer the RNA/DNA hybrid into a new well. *This is a safe stopping point. If needed, the plate can be stored at* ***-80°***

1. **Second Strand cDNA: Reaction Brew**
   1. Generate the “**SS_cDNA_2nd_Strand**” brew using LIMS.

LIMS: Prepare Standard Solutions > SS_cDNA_2nd_Strand > follow the prompts > Save Standard Solution

- 1. Retrieve 1D Large label from 5^th^ floor printer outside the RNA room and brew mix check-list label from RNA Room printer.
  2. If LIMS is down, enter the number of samples to be processed and print the ss-cDNA Worksheet located in:

| R:\Lib core\Work Sheets and Calculators\Strand Specific\Manual\Manual Plate-based Strand-specific cDNA synthesis with Maxima H Minus |
| --- |

- 1. Prepare Strand specific 2^nd^ strand cDNA synthesis brew following the printed LIMS calculator or following the printed Excel spreadsheet. Make sure to mix each reagent well before adding to the brew. Add enzymes last.
  2. The reaction set up for 1 reaction is as follows:

| **Solution** | **1 rxn (µL)** |
| --- | --- |
|  |  |
| First Strand sscDNA | 35 |
|  |  |
| DEPC-dH2O | 1 |
| 5X Second Strand Buffer | 10 |
| GeneAmp mix with dUTP (12.5mM) | 1.5 |
| E.Coli DNA Ligase (10U/µL) | 0.5 |
| E.Coli DNA Polymerase (10U/µL) | 1.5 |
| E.Coli DNA RNase H (2U/µL) | 0.5 |
|  |  |
| **Reaction volume** | **50** |

Second Strand Brew Mix (15µL)

- 1. Make sure to mix each reagent well and quick spin before adding to the brew. Add enzymes last.
  2. Prepare the reaction brew and check off reagents as they are added. Mix the brew by repeated pulse-vortexing followed by a quick spin. Label the brew as SS Brew and place on ice until ready to use.
  3. Add 15 µL of the SS brew into each well of a new plate.
  4. Transfer the purified 1^st^ strand cDNA/mRNA hybrid into the corresponding wells of the plate containing the 2^nd^ strand reaction brew, mix 10 times, seal plate with VWR foil seal and spin down at 4°C, 2000g for 1min.
  5. In the Tetrad thermocycler, incubate the plate at 16°C for 2h 15min.

TETRAD: SDWTSS folder > cDNA_2

- 1. After thermo-cycler program is finished, remove the plate and spin it at 4°C, 2000g for 1min.

**Note: *This is a safe stopping point. If needed, the plate can be stored at -20°C, or on the tetrad overnight if there is insufficient time to take the plate out for cold storage***.

1. **Post–Second Strand cDNA: Ampure XP bead clean-up of DS cDNA**
   1. The input volume for this step is 50µL per well.
   2. To clean up cDNA after second strand reaction using Ampure XP beads, follow the modifications below and refer to:

LIBPR.0073- Manual Bead Clean using Ampure XP Beads

| **Reaction** | **Ampure XP bead* Vol (µL)** | **Bead Binding Time (mins)** | **Magnet Clearing Time (mins)** | **2X 70% EtOH* Wash Vol (µL)** | **Magnet Airdry Time (mins)** | **Elution Vol (µL)** | **Elution time (mins)** | **Magnet Elution time (mins)** | **Transfer Vol (µL)** |
| --- | --- | --- | --- | --- | --- | --- | --- | --- | --- |
| **2^nd^ strand**  **ss-cDNA** | **100** | **15** | **7** | **150** | **5** | **42** | **3** | **2** | **41 (1µL for QC)** |

*Must be at Room Temp for a minimum of 15mins before usage; failure to do so would result in decreased yield.

- - 1. It is critical to let the beads bind and separate on the magnet for a full 15 minutes and 7 minutes respectively. Removing the binding buffer before the beads have completely separated will impact cDNA yield.
    2. After the Magnet Elution time, transfer the RNA/DNA hybrid into a new well and remove 1µL for HS Agilent cDNA QC for all samples including controls.

1. **HS Agilent QC of DS cDNA**
   1. For quality and quantity control check of the cDNA use 1µL of each sample, including the controls for High Sensitivity DNA Agilent assay, according to protocol:

LIBPR.0017- Operation and Maintenance of the Agilent 2100 Bioanalyzer for DNA samples


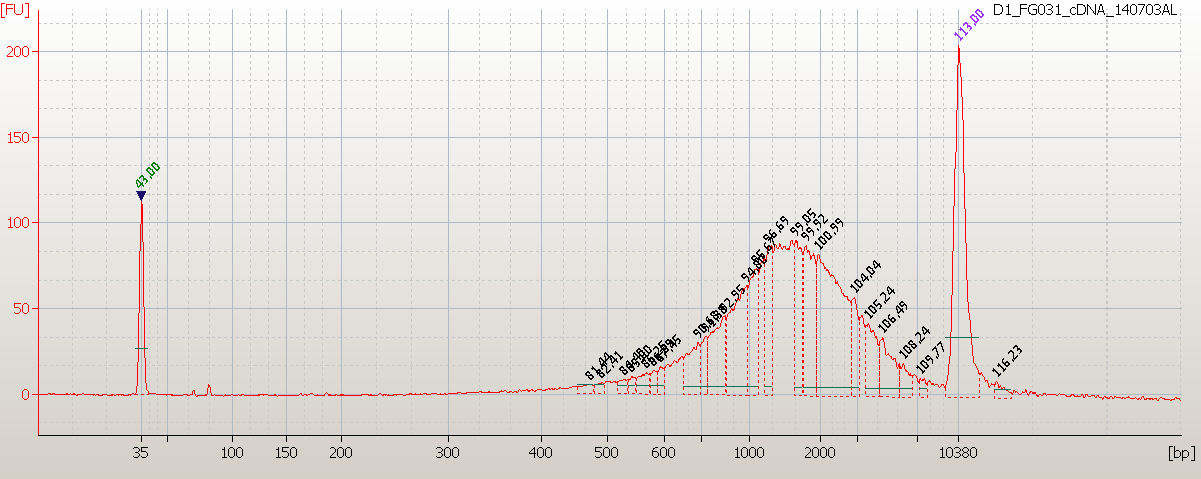


An example of the expected cDNA profile (from 2µg UHR total RNA). The FU levels should be viewed with caution here as levels are beyond the quantitative range, and thus greater fluctuations due to inherent QC variability are expected. Beyond QC related factors, cDNA amount is expected to vary depending on input Total RNA amount and quality, mRNA content, etc.

**Appendix A: LIMS**

## M-Strand Specific cDNA Synthesis: RNA_strategy is ‘strand specific’ and the pipeline will be SSTRA_2.1: Strand Specific Transcriptome 2.1

- Bioanalyzer Run – cDNA QC. There are no attributes to add. Approve the runs.

**Appendix B: Actinomycin D**

1. Actinomycin D is toxic and gloves must be worn when handling it.
2. Actinomycin D powder is hygroscopic and sensitive to light. When stored in original vial, manufacture sealed and protected from light and moisture, at 4°C, it remains unchanged for the amount of time specified by the manufacture (it is shown on the label). When receiving Actinomycin D powder in LIMS enter that date as the expiration date of the powder.
3. Dilute solutions of Actinomycin D are very sensitive to light. This product tends to adsorb to plastic and glass on standing in solution. It is important to take the aliquots out of the freezer only shortly before you are ready to use it, and after thawing using ActD solution quickly. Once taken out from the freezer, an aliquot should either be used up or discarded – it should never be re-frozen for later use.
4. The shelf life of re-suspended Actinomycin D when kept at 4°C and above is only few hours. It is therefore important to perform the re-suspension procedure in an efficient and quick manner.
5. Protected from light, frozen aliquots of 10µg/µL (or 10mg/mL which is 8mM) are expected to be stable up to 3 months at -20°C. After 3 months, all unused tubes of re-suspended, frozen Actinomycin D should be discarded and a new re-suspension batch should be made.
6. Preparation of 10µg/µL (or 10mg/mL) storage stock of Actinomycin D.
   1. Log into LIMS. Go to “Solutions” page. Under “Mix Standard Solutions” select **Actinomycin 10µg/µL** and 1 x 1 for samples (see image below). Click “Mix Standard Solution” button.


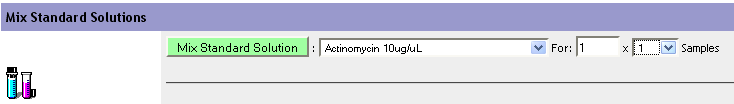


Figure 1: Mix Standard Solution

- 1. In the Parameters section enter the mg of Actinomycin D in the vial (see image below). Click “Re-calculate Standard Solution”.


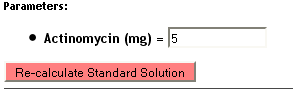


Figure 2: Re-calculate Standard Solution

- 1. In the Other Parameters section enter “Reagent” for Type, expiration date for 3 months ahead, select “Lib Construction” group, 2D solution labels. Click on the “Save Standard Mixture” button (see image below):


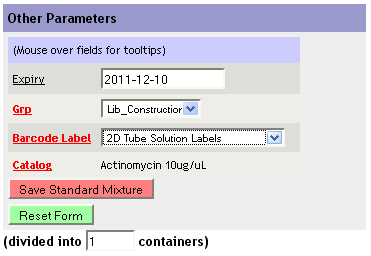


Figure 3: Save Standard Mixture

- 1. Retrieve both the solution barcode and the calculator from the corresponding printers.
  2. Using the newly generated barcode perform an aliquot step in LIMS dividing the total volume into 15µL per tube. Select 2D solution barcodes.

1. Add the amount of room temperature DMSO listed on the large LIMS calculator to the amber vial containing 5mg of Actinomycin D powder. Close the vial.
2. Mix well few times by gentle pulse-vortexing. Make sure that the powder is fully re-suspended.
3. Open the vial and mix by pipetting using P200.
4. Using 1.5mL amber tubes immediately aliquot Actinomycin D solution 15µL per tube. Place the tubes on ice.
5. Attach 2D solution barcodes generated in step 6.5 on the tubes.
6. Cover each tube with parafilm creating tight seal.
7. As soon as possible place tubes in 9x9 box, protected from light, in the -20°C freezer.
