# Supplementary material for: Increasing quality, throughput and speed of sample preparation for strand-specific messenger RNA sequencing
Source: BMC Genomics. 2017 Jul 5;18:515. doi: 10.1186/s12864-017-3900-6 (PMC5499059; doi:10.1186/s12864-017-3900-6)
Supplement: Supplementary file 3 — library construction. (DOCX 129 kb) [file 12864_2017_3900_MOESM3_ESM.docx]

**Manual PCR-enriched Library Construction for Illumina Sequencing**

# Purpose

To provide specific guidelines for Manual PCR-enriched ss-cDNA/DNA library construction for Illumina Paired-End Sequencing

# Scope

All procedures are applicable to the BCGSC Library core and Library TechD groups.

# Policy

This procedure will be controlled under the policies of the Genome Sciences Centre, as outlined in the Genome Sciences Centre High Throughput Production Quality Manual (QM.0001). Do not copy or alter this document. To obtain a copy see a QA associate.

# Responsibility

It is the responsibility of all personnel performing this procedure to follow the current protocol. It is the responsibility of the Group Leader to ensure personnel are trained in all aspects of this protocol. It is the responsibility of Quality Assurance Management to audit this procedure for compliance and maintain control of this procedure.

# References

| Document Title | Document Number |
| --- | --- |
| Sample Preparation for Paired-End Sample Prep Kit from Illumina | Version 1.1 (from Prep Kit) |

# Related Documents

| Document Title | Document Number |
| --- | --- |
| 96-well DNA Quantification using the dsDNA Quant-iT High Sensitivity Assay Kit and VICTOR3V | LIBPR.0108 |
| Operation of the Covaris LE220 | LIBPR.0097 |
| Operation and Maintenance of the Agilent 2100 Bioanalyzer for DNA samples | LIBPR.0017 |
| Operation and Maintenance of the Caliper Labchip GX for DNA samples using the High Sensitivity Assay | LIBPR.0051 |
| Operation of the Invitrogen Egel iBase Power System | LIBPR_WORKINST.0012 |
| Quantifying DNA samples using the Qubit Fluorometer | LIBPR.0030 |
| Span-8 Pooling of DNA Samples | LIBPR.0093 |
| Manual Bead Clean Up using Ampure XP Beads | LIBPR.0073 |

# Safety

All Laboratory Safety procedures will be complied with during this procedure. The required personal protective equipment includes a laboratory coat and gloves. See the material safety data sheet (MSDS) for additional information.

# Materials and Equipment

| Name | Supplier | Number: # | Model or Catalogue # | |
| --- | --- | --- | --- | --- |
| NEB Paired-End Sample Prep Premix Kit – End Repair | NEB | E6875B-GSC |  |  |
| NEB Paired-End Sample Prep Premix Kit – A Tail | NEB | E6876B-GSC |  |  |
| NEB Paired-End Sample Prep Premix Kit – Ligation | NEB | E6877B-GSC |  |  |
| Phusion Hotstart | Fisher | F540L |  |  |
| Fisherbrand Textured Nitrile gloves – various sizes | Fisher | 270-058-53 |  |  |
| Ice bucket – Green | Fisher | 11-676-36 |  |  |
| Wet ice | In house | N/A | N/A | N/A |
| Covaris E210 | Transition Technologies Inc. | E210 |  |  |
| DNA *AWAY* | Molecular BioProducts | 21-236-28 |  |  |
| AB1000 PCR plate | Fisher Scientific | AB1000 |  |  |
| Gilson P2 pipetman | Mandel | GF-44801 |  |  |
| Gilson P10 pipetman | Mandel | GF-44802 |  |  |
| Gilson P20 pipetman | Mandel | GF23600 |  |  |
| Gilson P200 pipetman | Mandel | GF-23601 |  |  |
| Gilson P1000 pipetman | Mandel | GF-23602 |  |  |
| Diamond Filter tips DFL10 | Mandel Scientific | GF -F171203 |  |  |
| Diamond Filter tips DF30 | Mandel Scientific | GF-F171303 |  |  |
| Diamond Filter tips DF200 | Mandel Scientific | GF-F171503 |  |  |
| Diamond Filter tips DF1000 | Mandel Scientific | GF-F171703 |  |  |
| Galaxy mini-centrifuge | VWR | 37000-700 |  |  |
| VX-100 Vortex Mixer | Rose Scientific | S-0100 |  |  |
| Black ink permanent marker pen | VWR | 52877-310 |  |  |
| Clear Tape Sealer | Qiagen | 19570 |  |  |
| Aluminum Foils seals for UNG digestion | VWR | 60941-126 |  | 🗸 |
| Aluminum foil tape, 3"x 60 yds | Scotch/3M | 34000740 |  | 🗸 |
| Eppendorf BenchTop Refrigerated Centrifuge 5810R | Eppendorf | 5810 R |  |  |
| Bench Coat (Bench Protection Paper) | Fisher | 12-007-186 |  |  |
| Small Autoclave waste bags 10”X15” | Fisher | 01-826-4 |  |  |
| Anhydrous Ethyl Alcohol (100% Ethanol) | CommercialAlcohols | 00023878 |  |  |
| IKA Works Vortexer | Agilent | MS2S9-5065-4428 |  |  |
| 22R Microfuge Centrifuge | Beckman | 22R Centrifuge |  |  |
| Peltier Thermal Cycler | MJ Research | PTC-225 |  |  |
| Power Supply, LVC2kW, 48VDCV | Tyco Electronics | RM200HA100 |  |  |
| Eppendorf Benchtop Centrifuge | Eppendorf | 5810 R |  |  |
| 70% Ethanol | In house | N/A | N/A | N/A |
| Qiagen Buffer EB – 250 mL | Qiagen | 19086 |  |  |
| 10 X TBE - 10 L | Invitrogen | 15581-028 |  |  |
| 1 X TBE | In House | N/A | N/A | N/A |
| UltraPure Distilled Water | Invitrogen | 10977-023 |  |  |
| Uracil N-Glycosylase (UNG) | Applied Biosystems | N808-0096 |  |  |
| PCR Clean DX (ALINE beads) | ALINE Biosciences | C-1003-450 |  |  |
| USER Enzyme | NEB | M5505L |  |  |

**These sequences are for internal use only:**

**PE adapters:**

5’ ACACTCTTTCCCTACACGACGCTCTTCCGATCT

3’ GAGCCGTAAGGACGACTTGGCGAGAAGGCTAG

**PE PCR Primers**

5' AATGATACGGCGACCACCGAGATCTACACTCTTTCCCTACACGACGCTCTTCCGATCT

5'CAAGCAGAAGACGGCATACGAGATNNNNNNCGGTCTCGGCATTCCTGCTGAACCGCTCTTCCGATCT

# Introduction and Guidelines

## General Guidelines

- 1. Ensure proper personal protective equipment is used when handling sample plates, reagents and equipment. Treat everything with clean PCR techniques.
  2. Wipe down the assigned workstation, pipetman, tip boxes and small equipment with DNA *AWAY*. Ensure you have a clean working surface before you start.
  3. Pre-PCR and Post-PCR work should be performed in their designated locations.
  4. Acronyms: NA stands for Not Applicable. Pre-LC refers to Pre-Library Construction. Post-LC refers to Post-Library Construction. BC refers to Bead Clean.
  5. Color code: red fonts designate exceptions or protocol-specific steps.
  6. Discuss with the APC/PC/designated trainer the results of every QC step. Report and record equipment failures and/or malfunctions and variations in reaction well volumes.

## General Plate Guidelines

- 1. To avoid cross-well contamination, reaction plates should never be vortexed and plate seals should never be re-used.
  2. Use Qiagen tape seals for short term storage, VWR foil seals for tetrad incubations/UNG digestion/PCR, and 3M aluminum foil seal for long term storage.
  3. After completion of every incubation step, quick spin the plate(s) at 4ºC for 1 minute at 2000 g.
  4. Sample plates can be stored at -20ºC overnight after every step except post “A” addition. **“A” addition and adapter ligation reactions must be performed on the same day.**

## Positive and Negative Controls

- 1. The positive control template to be used for this protocol is HL60 genomic DNA or UHR cDNA. The yield of library products constructed from positive controls is expected to differ from those of Collaborators’ samples. However, the yield should not differ significantly from that of previously constructed positive controls.
  2. The negative control template to be used for this protocol is Qiagen Elution Buffer. This control will ensure the absence of background products that result from the library construction process.

## General Brew Preparation Guidelines

- 1. Double check the QA release and expiry date of each reagent and enzyme.
  2. Thaw required reagents and place them on ice. Enzymes should be left in the freezer until ready to use.
  3. Reagents and enzymes should be well mixed, the former by pulse-vortexing and the latter by gentle flicking. After mixing, quick spin down in a mini-centrifuge.
  4. All premixed and prepared brews should be well mixed by gentle, repeated pulse-vortexing to ensure equal distribution of all components and thus uniformity of enzymatic reactions across a plate. The End-Repair and Ligation brews are particularly viscous.
  5. All reactions require the preparation of a Brew Source Plate.
  6. All brew calculators include excess volume to account for dead volume to account for pipetting loss.

## General note on bead clean up module:

The bead cleanup modules employed in this SOP are based on the following conditions:

| **Bead Binding Time (mins)** | **1^st^ Magnet Clearing Time (mins)** | **2 X 70% EtOH Wash Vol (µL)** | **Ethanol Air-dry Time (mins)** | **Elution Volume (µL)** | **Elution time (mins)** | **2^nd^ Magnet Clearing time (mins)** |
| --- | --- | --- | --- | --- | --- | --- |
| **15** | **7** | **150** | **5** | **20-52** | **3** | **2** |

Note: Bead to reaction ratio are 1.8:1 for post-shearing, 2:1 for other pre-ligation clean ups and 1:1 for post-ligation reactions.

The elution volumes are specified below for each of the steps:

| **Step** | **Elution Volume (µL)** |
| --- | --- |
| Post-End repair | 32 |
| Post-A addition | N/A |
| Post-ligation 1^st^ cleanup | 52 |
| Post-ligation 2^nd^ cleanup | 20 |
| Post-iPCR 1^st^  cleanup | 52 |
| Post-iPCR 2^nd^  cleanup | 25 |

# Procedure

Note: ALINE beads can be used as a direct replacement of Ampure XP magnetic beads in steps that specify the use of Ampure XP magnetic beads.

## Initial QC

- 1. For each gDNA 96 well stock plate, quantify according to:

| LIBPR.0030- Quantifying DNA samples using the Qubit Fluorometer |
| --- |

Note: this does not apply to cDNA or ChIP DNA.

- 1. This protocol is designed to work with a range of input specified below:

| **Starting Material** | **Amount (µg)** |
| --- | --- |
| ss-cDNA | NA |
| Small gap gDNA | 0.5-1.0 |
| ChIP DNA | NA |

## Shearing

Refer to the following instructions for shearing:

| LIBPR.0097 Operation of the Covaris LE220 |
| --- |

Note: this does not apply to ChIP DNA.

**Make sure that you have performed the shearing twice with a spin in between according to the SOP above.**

## Agilent HS DNA QC after shearing – Spot Check

- 1. Use 1 µL from 11 random samples (ensure that at least one of these samples is a positive control) to spot check on a High Sensitivity DNA Agilent Assay according to protocol:

| LIBPR.0017 Operation and Maintenance of the Agilent 2100 Bioanalyzer for DNA samples |
| --- |

- 1. The following table shows the expected average size from the sheared material:

| **Starting Material** | **Average size** |
| --- | --- |
| ss-cDNA | 200-250bp |
| Small gap | 250bp |

Note: For ribo-depleted ss-cDNA, the products will not be visible on the HSDNA chip. Use the non-depleted UHR control to QC accurate shearing.

## Post-shearing cleanup

Post-shearing cleanup is for Small gap only. This does not apply to ss-cDNA or ChIP DNA.

- 1. Clean up sheared Small Gap gDNA as described in the following SOP:

| LIBPR.0073- Manual Bead Clean using Ampure XP Beads |
| --- |

- 1. Specific volumes are highlighted below.

| **DNA volume (µL)** | **Bead Volume (µL)** | **Mixing Volume (µL)** | **Bead Binding Time (mins)** | **Magnet Clearing Time (mins)** | **Supernatant Volume (µL)** | **2x 70% EtOH Wash Vol (µL)** | **Ethanol Air Dry Time (mins)** | **EB Elution Volume (µL)** | **Elution Time (mins)** | **Magnet Elution Time (mins)** | **Transfer Volume (µL)** |
| --- | --- | --- | --- | --- | --- | --- | --- | --- | --- | --- | --- |
| 60 | 108 | 135 | 15 | 7 | 168 | 150 | 5 | 37 | 3 | 2 | 35 |

## End-Repair and Phosphorylation Reaction

- 1. The volume requirement for 1 reaction set up is as follows:

| **Solution** | **1 rxn (µL)** |
| --- | --- |
| DNA | 35 |
| NEB End Repair Premix | 23.5 |
| **Reaction volume** | **58.5** |

- 1. Dispense **23.5 µL of End Repair Premix** into each well of a destination plate.
  2. Transfer DNA to each well of the destination plate containing the reaction brew. Mix 10 times. Cover with plate seal and quick spin at 4ºC for 1 minute.
  3. Incubate End-Repair reaction plate at 20ºC for 30 minutes.

**Tetrad Program: Run > LIBCOR > ER**

## Magnetic Bead Clean Up after End-Repair

- 1. The input volume for this step is 58.5 µL per well.
  2. Clean up End Repaired DNA using Magnetic beads as described in the following SOP:

| LIBPR.0073- Manual Bead Clean using Ampure XP Beads |
| --- |

- 1. Specific volumes are highlighted below.

| **DNA volume (µL)** | **Bead Volume (µL)** | **Mixing Volume (µL)** | **Bead Binding Time (mins)** | **Magnet Clearing Time (mins)** | **Supernatant Volume (µL)** | **2x 70% EtOH Wash Vol (µL)** | **Ethanol Air Dry Time (mins)** | **EB Elution Volume (µL)** | **Elution Time (mins)** | **Magnet Elution Time (mins)** | **Transfer Volume (µL)** |
| --- | --- | --- | --- | --- | --- | --- | --- | --- | --- | --- | --- |
| 58.5 | 105 | 131 | 15 | 7 | 163.5 | 150 | 5 | 32 | 3 | 2 | 30 |

**Note that end repaired product can be stored at -20^o^C after the bead cleanup**.

## Addition of an ‘A’ Base (A-Tailing) Reaction

- 1. The volume requirement for 1 reaction set up is as follows:

| **Solution** | **1 rxn (µL)** |
| --- | --- |
| End-Repair + BC DNA | 30 |
| NEB Adenylation Premix | 20 |
| **Reaction volume** | **50** |

- 1. Dispense **20 µL of Adenylation Premix** into each well of a destination plate. Cover with plate seal and quick spin at 4ºC for 1 minute.
  2. Transfer DNA to each well of the destination plate containing the reaction brew. Mix 10 times. Cover with plate seal and quick spin at 4ºC for 1 minute.
  3. Incubate A-tailed reaction plate(s) at 37ºC for 30 minutes, 70 ºC for 5 minutes, 4 ºC for 5 minutes; hold at 4 ºC using the following tetrad program:

**Tetrad Program: Run > LIBCOR > ATAIL**

- 1. Once the plate is held at 4C, proceed immediately to the next step. This is NOT a safe stopping point. **Ligation is performed in-tandem immediately after the A tail reaction. Store adenylated products temporarily on ice while preparing the ligation reaction.**

## Illumina PE Adapter Ligation Reaction

- 1. Thaw the PE Adapter stock aliquot at a designated Pre-PCR work bench then immediately place on ice.
  2. Adapter Ligation brew (minus the PE adapter) must be made in a designated laminar flowhood. Add PE adapter to the brew at a designated pre-PCR work bench.
  3. The volume requirement for 1 reaction for SSTRA_2.2, Ribodepletion and ChIP is as follows:

**Note: for SSTRA_3.0, refer to expert protocol in Appendix C for ligation calculator.**

| **Solution** | **1 rxn (µL)** |
| --- | --- |
| A-Tail reaction | 50 |
| 2X NEB Ligation Premix | 21  Ligation_Brew_10pmol  (25µL) |
| dH_2_O | 3 |
| PE Adapter (10 µM) | 1 |
| **Reaction volume** | **75** |

- 1. The volume requirement for 1 reaction for Small Gap is as follows:

| **Solution** | **1 rxn (µL)** |
| --- | --- |
| A-Tail reaction | 50  Ligation_Brew_40pmol  (25µL) |
| 2X NEB Ligation Premix | 21 |
| PE Adapter (10 µM) | 4 |
| **Reaction volume** | **75** |

- 1. Generate the Ligation-Brew Mix calculator using LIMS:

| LIMS: Mix Standard Solutions > X > *follow the prompts*> Save Standard Solution |
| --- |

*X=Ligation_Brew_10pmol or Ligation_Brew_40pmol

- 1. To minimize adapter-adapter ligation, work quickly on ice and proceed as follows:
     1. Prepare the Ligation brew in an appropriate sized tube according to the chemistry calculator.
     2. Add the PE adapter to the brew last, not more than 10min before brew addition to DNA. Make sure the brew is on ice all the time.
     3. Immediately after the brew is prepared, dispense 25 µL of brew into each well of a destination plate.
     4. Transfer DNA to each well of the destination plate containing the reaction brew. Mix 10 times. Cover with plate seal and quick spin at 4ºC for 1 minute.
     5. Cover the brew source plate with plate seal and quick spin at 4ºC for 1 minute.
     6. Keep plates on ice but *proceed quickly* to the next step.
  2. Incubate Adapter Ligat*ion reaction pl*ate(s) at 20ºC for 15 minutes.

**Tetrad Program: Run > LIBCOR > LIGATION**

## Magnetic Bead Clean Up after Adapter Ligation

- 1. Clean up ligated DNA using Magnetic beads as described in the following SOP.

| LIBPR.0073- Manual Bead Clean using Ampure XP Beads |
| --- |

- 1. Specific volumes Specific volumes are highlighted below. Note that two bead cleans are performed post ligation. Note that the EB elution volumes are different for the first and second bead clean.

**Bead clean #1**

| **DNA volume (µL)** | **Bead Volume (µL)** | **Mixing Volume (µL)** | **Bead Binding Time (mins)** | **Magnet Clearing Time (mins)** | **Supernatant Volume (µL)** | **2x 70% EtOH Wash Vol (µL)** | **Ethanol Air Dry Time (mins)** | **EB Elution Volume (µL)** | **Elution Time (mins)** | **Magnet Elution Time (mins)** | **Transfer Volume (µL)** |
| --- | --- | --- | --- | --- | --- | --- | --- | --- | --- | --- | --- |
| 75 | 75 | 120 | 15 | 7 | 150 | 150 | 5 | 52 | 3 | 2 | 50 |

**Bead clean #2**

| **DNA volume (µL)** | **Bead Volume (µL)** | **Mixing Volume (µL)** | **Bead Binding Time (mins)** | **Magnet Clearing Time (mins)** | **Supernatant Volume (µL)** | **2x 70% EtOH Wash Vol (µL)** | **Ethanol Air Dry Time (mins)** | **EB Elution Volume (µL)** | **Elution Time (mins)** | **Magnet Elution Time (mins)** | **Transfer Volume (µL)** |
| --- | --- | --- | --- | --- | --- | --- | --- | --- | --- | --- | --- |
| 50 | 50 | 80 | 15 | 7 | 100 | 150 | 5 | 20 | 3 | 2 | 19 |

## Indexed PCR Amplification Reaction OR UNG digestion with PCR for SSTRA_2.2 and Ribodepletion

- 1. Thaw the PE PCR primer 1.0 and indexing primer plate at a designated Pre-PCR work bench then immediately place on ice. Quick spin the indexing primer plate prior to taking an aliquot.
  2. To keep track of freeze-thaw cycles, mark off the indexing primer plate each time the plate is thawed even if it is not used.
  3. The maximum freeze-thaw cycles for the indexing primer plate are 5 times.
  4. iPCR brew (minus the primers) must be in a designated Pre-PCR laminar flowhood. Add PE PCR primer 1.0 to the brew at a designated pre-PCR work bench.
  5. The volume requirement for 1 reaction set up for ChIP and Small gap are as follows:

| **Solution** | **1 rxn (µL)** |
| --- | --- |
| Adapter Ligated + BC DNA (**full-template**) | 19 |
| 5X Phusion HF Buffer | 10 |
| 10mM dNTP | 1  PCR Brew  (27µL) |
| DMSO | 1.5 |
| Hot Start Phusion (2 U/µL) | 0.5 |
| PE PCR primer 1.0 (25 µM) | 2 |
| dH_2_O | 12 |
| Indexed PCR primer plate (12.5µM) | 4 |
| **Reaction volume** | **50** |

- 1. The volume requirement for SSTRA_2.2 and Ribodepletion is as follows:

**Note: for SSTRA_3.0, refer to expert protocol in Appendix C for PCR Brew calculator.**

| **Solution** | **1 rxn (µL)** |
| --- | --- |
| Adapter Ligated + BC DNA (**full-template**) | 19 |
| 5X Phusion HF Buffer | 10 |
| 10mM dNTP | 1  PCR Brew + UNG  (27µL) |
| DMSO | 1.5 |
| Hot Start Phusion (2 U/µL) | 0.5 |
| PE PCR primer 1.0 (25 µM) | 2 |
| UNG | 5 |
| dH_2_O | 7 |
| Indexed PCR primer plate (12.5µM) | 4 |
| **Reaction volume** | **50** |

- 1. Generate the PCR Brew Mix calculator using LIMS:

| LIMS: Mix Standard Solutions > **LibConst_IndexingPCR_Brew**  **OR LibConst_IndexingPCR plus UNG_Brew** > *follow the prompts* > Save Standard Solution |
| --- |

- 1. Obtain the 1D large Solution/Box/Kit Label and Chemistry Label. Prepare the brew in an appropriate sized tube according to the chemistry calculator.
  2. Dispense 4 µL of Index primer into each well of a destination plate.
  3. Add 27 µL of PCR reaction brew into each well of the destination plate containing the index primers.
  4. Transfer 19 µL of DNA (all) into each well of the destination plate containing the brew and index primers. Mix 10 times. Cover with plate seal and quick spin at 4ºC for 1 minute.
  5. Run PCR program specified in the table below. Use a rubber pad on top of the reaction plate.

PCR parameters for ss-cDNA:

- 37˚C 15 min
- 98˚C 1 min
- 98˚C 15 sec

*Total of 10 or 13 Cycles

- 65˚C 30 sec
- 72˚C 30 sec
- 72˚C 5min
- 4˚C ∞

PCR parameters for others:

- 98˚C 1 min
- 98˚C 15 sec

*Total of 6 or 13 Cycles

- 65˚C 30 sec
- 72˚C 30 sec
- 72˚C 5min
- 4˚C ∞

*The number of PCR cycles is dependent on each of the protocol:

| **Starting Material** | **PCR cycles** | **Tetrad Program** |
| --- | --- | --- |
| ss-cDNA (poly-A) mRNA **(SSTRA_2.2)**  ss-cDNA (ribo-depleted) | 10  13 | SSCDNA10  SSCDNA13 |
| Small gap/Amplicon | 6 | LCPCR-6 |
| ChIP DNA | 13 | LCPCR-13 |

**Note: for SSTRA_3.0, refer to expert protocol in Appendix C for PCR cycles.**

1. **Post-LC Size Selection**
   1. Clean up PCR-enriched template using Magnetic beads as described in the following SOP:

| LIBPR.0073- Manual Bead Clean using Ampure XP Beads |
| --- |

- 1. Specific volumes are highlighted below. Note that two bead clean up reactions are performed post PCR amplification.

**Bead clean #1**

| **DNA volume (µL)** | **Bead Volume (µL)** | **Mixing Volume (µL)** | **Bead Binding Time (mins)** | **Magnet Clearing Time (mins)** | **Supernatant Volume (µL)** | **2x 70% EtOH Wash Vol (µL)** | **Ethanol Air Dry Time (mins)** | **EB Elution Volume (µL)** | **Elution Time (mins)** | **Magnet Elution Time (mins)** | **Transfer Volume (µL)** |
| --- | --- | --- | --- | --- | --- | --- | --- | --- | --- | --- | --- |
| 50 | 50 | 80 | 15 | 7 | 100 | 150 | 5 | 52 | 3 | 2 | 50 |

**Bead clean #2**

| **DNA volume (µL)** | **Bead Volume (µL)** | **Mixing Volume (µL)** | **Bead Binding Time (mins)** | **Magnet Clearing Time (mins)** | **Supernatant Volume (µL)** | **2x 70% EtOH Wash Vol (µL)** | **Ethanol Air Dry Time (mins)** | **EB Elution Volume (µL)** | **Elution Time (mins)** | **Magnet Elution Time (mins)** | **Transfer Volume (µL)** |
| --- | --- | --- | --- | --- | --- | --- | --- | --- | --- | --- | --- |
| 50 | 50 | 80 | 15 | 7 | 100 | 150 | 5 | 25 | 3 | 2 | 23 |

- 1. Thoroughly mix the beads before the second bead clean up. Note that the libraries can be stored at -20^o^C after the 1^st^ or 2^nd^ bead cleanup.

## Final QC

LIBPR.0030 Quantifying DNA Samples using the Qubit Fluorometer

## Final Agilent DNA1000 QC

| LIBPR.0017-Operation and Maintenance of the Agilent 2100 Bioanalyzer for DNA samples |
| --- |

## Pool samples into 1.5mL tubes (if needed) or rearray unpooled samples into 1.5mL tubes

## QC of pooled samples

## Quantify pooled samples using Qubit:

LIBPR.0030 Quantifying DNA Samples using the Qubit Fluorometer

1. **Sequencing Submission**

For each library, determine the corrected final molar concentration for submission to sequencing. Use the average base pair size previously obtained from the Agilent profile and the result from the Qubit quantification to obtain the final size-corrected concentration (nM). Remaining final volume (after QCs) is ~20uL.

**Appendix A.: LIMS Protocol**

- - - 1. Start of Plate Library Construction – IDX pipeline (skip if doing ss-cDNA)
      2. Bioanalyzer Run – QC Category: Sonication QC (skip if doing ChIP)
      3. M-Library Construction – IDX pipeline
      4. Plate_Indexed_PCR – IDX pipeline
      5. Plate_PPBC_SizeSelection – IDX pipeline
      6. Bioanalyzer Run – QC Category: Post library construction size selection

**Note: for libraries going into multiplex capture, please select “Post-PCR QC” as the QC Category instead. No need to enter attributes and please skip remaining steps.**

Enter the following attributes:

- - - Library_size_distribution_bp (From Agilent)
    - Avg_DNA_bp_size (From Agilent)
    - DNA_concentration_ng_µL (From Qubit)
      1. If pooling: Aliquot volume for pooling into a new tra
      2. If pooling: Pooling and/or Manual Rearray into tubes – IPE or PET pipeline
      3. Final_Submission – IPE or PET pipeline

**Appendix B: Expert Protocol: Manual PCR-enriched Library Construction**

**Applicable to Small Gap, SSTRA_2.2, Ribodepletion and ChIP**

1. **Shearing & QC (ss-cDNA, and Small gap only)**
   1. Transfer all ss-cDNA or gDNA, to Covaris LE220 tubes
   2. Covaris LE220, LIBPR.0097
   3. QC: Agilent HS DNA Assay
2. **Bead Clean Sheared gDNA (Small Gap only)**
   1. Ethanol and Magnetic beads must be incubated at room temperature for at least 30 minutes before use.

| **DNA volume (µL)** | **Bead Volume (µL)** | **Mixing Volume (µL)** | **Bead Binding Time (mins)** | **Magnet Clearing Time (mins)** | **Supernatant Volume (µL)** | **2x 70% EtOH Wash Vol (µL)** | **Ethanol Air Dry Time (mins)** | **EB Elution Volume (µL)** | **Elution Time (mins)** | **Magnet Elution Time (mins)** | **Transfer Volume (µL)** |
| --- | --- | --- | --- | --- | --- | --- | --- | --- | --- | --- | --- |
| 60 | 108 | 135 | 15 | 7 | 168 | 150 | 5 | 37 | 3 | 2 | 35 |

1. **End Repair & Phosphorylation**

| **Solution** | **1 rxn (µL)** |
| --- | --- |
| DNA | 35 |
| NEB End Repair Premix | 23.5 |
| **Reaction volume** | **58.5** |

- 1. Transfer 23.5 µL of NEB End Repair Premix into wells of a destination plate.
  2. Transfer 35 µL of ss-cDNA, ChIP DNA or gDNA to End Repair Premix, mix using 80% volume, 10X.
  3. Tetrad Program: LIBCOR>ER 20C, 30 min; hold 4C
  4. Safe stopping point if stored at -20C.

1. **Bead Clean End Repaired & Phosphorylated Template**
   1. Ethanol and Magnetic beads must be incubated at room temperature for at least 30 minutes before use.

| **DNA volume (µL)** | **Bead Volume (µL)** | **Mixing Volume (µL)** | **Bead Binding Time (mins)** | **Magnet Clearing Time (mins)** | **Supernatant Volume (µL)** | **2x 70% EtOH Wash Vol (µL)** | **Ethanol Air Dry Time (mins)** | **EB Elution Volume (µL)** | **Elution Time (mins)** | **Magnet Elution Time (mins)** | **Transfer Volume (µL)** |
| --- | --- | --- | --- | --- | --- | --- | --- | --- | --- | --- | --- |
| 58.5 | 105 | 131 | 15 | 7 | 163.5 | 150 | 5 | 32 | 3 | 2 | 30 |

- 1. Note: This is a safe stopping point. Do not proceed to adenylation unless you have adequate time to perform ligation reaction as well.

**A-Tailing**

| **Solution** | **1 rxn (µL)** |
| --- | --- |
| End-Repair + BC DNA | 30 |
| NEB Adenylation Premix | 20 |
| **Reaction volume** | **50** |

- 1. Transfer 20 µL of NEB Adenylation Premix to 30µL of size selected and repaired/phosphorylated DNA.
  2. Tetrad Program: LIBCOR>ATAIL
  3. Proceed directly to in-tandem ligation (do not bead clean after Adenylation). Store on ice while preparing Ligation premix and adapters.

1. **Adapter Ligation**

Ligation_Brew_10 pmoles: ss-cDNA, ChIP only

| **Solution** | **1 rxn (µL)** |
| --- | --- |
| **Adenylated template** | **50** |
| **NEB Ultra Premix 2X** | 21  Ligation_Brew_10pmol  (25µL) |
| PE Adapter (10 µM) | 1 |
| dH2O | 3 |
| **Reaction volume** | **75** |

Ligation_Brew_40 pmoles: Small Gap only

| **Solution** | **1 rxn (µL)** |
| --- | --- |
| **Adenylated template** | **50**  Ligation_Brew_40pmol  (25µL) |
| **NEB Ultra Premix 2X** | 21 |
| PE Adapter (10 µM) | 4 |
| **Reaction volume** | **75** |

- 1. Transfer 25µL of ligation brew to 50µL of adenylated template.
  2. Reset pipette to 80% total volume, mix 10X.
  3. Select tetrad program: LIBCOR>LIGATION
  4. Set a timer for 15 minutes. Quick spin plate and store on ice immediately after the 15 minute ligation.

1. **Double Bead Clean post Ligation**

**Bead clean #1**

| **DNA volume (µL)** | **Bead Volume (µL)** | **Mixing Volume (µL)** | **Bead Binding Time (mins)** | **Magnet Clearing Time (mins)** | **Supernatant Volume (µL)** | **2x 70% EtOH Wash Vol (µL)** | **Ethanol Air Dry Time (mins)** | **EB Elution Volume (µL)** | **Elution Time (mins)** | **Magnet Elution Time (mins)** | **Transfer Volume (µL)** |
| --- | --- | --- | --- | --- | --- | --- | --- | --- | --- | --- | --- |
| 75 | 75 | 120 | 15 | 7 | 150 | 150 | 5 | 52 | 3 | 2 | 50 |

**Bead clean #2**

| **DNA volume (µL)** | **Bead Volume (µL)** | **Mixing Volume (µL)** | **Bead Binding Time (mins)** | **Magnet Clearing Time (mins)** | **Supernatant Volume (µL)** | **2x 70% EtOH Wash Vol (µL)** | **Ethanol Air Dry Time (mins)** | **EB Elution Volume (µL)** | **Elution Time (mins)** | **Magnet Elution Time (mins)** | **Transfer Volume (µL)** |
| --- | --- | --- | --- | --- | --- | --- | --- | --- | --- | --- | --- |
| 50 | 50 | 80 | 15 | 7 | 100 | 150 | 5 | 20 | 3 | 2 | 19 |

- 1. The ligated template can be stored at -20C after the first or second bead clean up step.

1. **PCR enrich adapter-ligated template**
   1. The volume requirement for 1 reaction set up for Small Gap and ChIP is as follows:

| **Solution** | **1 rxn (µL)** |
| --- | --- |
| Adapter Ligated + BC DNA | 19 |
| 5X Phusion HF Buffer | 10 |
| 10mM dNTP | 1  PCR Brew  (27µL) |
| DMSO | 1.5 |
| Hot Start Phusion (2 U/µL) | 0.5 |
| PE PCR primer 1.0 (25 µM) | 2 |
| dH_2_O | 12 |
| Indexed PCR primer plate (12.5µM) | 4 |
| **Reaction volume** | **50** |

- 1. The volume requirement for 1 reaction set up for ss-cDNA is as follows:

| **Solution** | **1 rxn (µL)** |
| --- | --- |
| Adapter Ligated + BC DNA | 19 |
| 5X Phusion HF Buffer | 10 |
| 10mM dNTP | 1  PCR Brew + UNG  (27µL) |
| DMSO | 1.5 |
| Hot Start Phusion (2 U/µL) | 0.5 |
| PE PCR primer 1.0 (25 µM) | 2 |
| UNG | 5 |
| dH_2_O | 7 |
| Indexed PCR primer plate (12.5µM) | 4 |
| **Reaction volume** | **50** |

- 1. Select Pipeline-specific tetrad program:

| **Starting Material** | **PCR cycles** | **Tetrad Program** |
| --- | --- | --- |
| ss-cDNA(poly-A)  ss-cDNA (ribo-depleted) | 10  13 | SSCDNA10  SSCDNA13 |
| Small gap/Amplicon | 6 | LCPCR-6 |
| ChIP DNA | 13 | LCPCR-13 |

- 1. PCR-enriched template can be stored at -20C or proceed immediately to bead clean PCR enriched template.

1. **Double Bead Clean post iPCR**

**Bead clean #1**

| **DNA volume (µL)** | **Bead Volume (µL)** | **Mixing Volume (µL)** | **Bead Binding Time (mins)** | **Magnet Clearing Time (mins)** | **Supernatant Volume (µL)** | **2x 70% EtOH Wash Vol (µL)** | **Ethanol Air Dry Time (mins)** | **EB Elution Volume (µL)** | **Elution Time (mins)** | **Magnet Elution Time (mins)** | **Transfer Volume (µL)** |
| --- | --- | --- | --- | --- | --- | --- | --- | --- | --- | --- | --- |
| 50 | 50 | 80 | 15 | 7 | 100 | 150 | 5 | 52 | 3 | 2 | 50 |

**Bead clean #2**

| **DNA volume (µL)** | **Bead Volume (µL)** | **Mixing Volume (µL)** | **Bead Binding Time (mins)** | **Magnet Clearing Time (mins)** | **Supernatant Volume (µL)** | **2x 70% EtOH Wash Vol (µL)** | **Ethanol Air Dry Time (mins)** | **EB Elution Volume (µL)** | **Elution Time (mins)** | **Magnet Elution Time (mins)** | **Transfer Volume (µL)** |
| --- | --- | --- | --- | --- | --- | --- | --- | --- | --- | --- | --- |
| 50 | 50 | 80 | 15 | 7 | 100 | 150 | 5 | 25 | 3 | 2 | 23 |

- 1. Template can be stored at -20C after the first or second bead clean up post PCR.

1. **QC Final Library Products**
   1. Run 1uL of each final library product on Agilent DNA 1000 chip assay.
   2. Quantify each final library product by Qubit HS DNA assay.
   3. If required, pool samples by equal molar pooling and quantify pool by Qubit HS DNA assay.

**Appendix C: Expert Protocol: Manual PCR-enriched Library Construction for Applicable to SSTRA_3.0 only**

1. **Shearing & QC**
   1. Transfer all ss-cDNA to Covaris LE220 tubes
   2. Covaris LE220, LIBPR.0097
   3. QC: Agilent HS DNA Assay
2. **End Repair & Phosphorylation**

| **Solution** | **1 rxn (µL)** |
| --- | --- |
| cDNA | 35 |
| NEB End Repair Premix | 23.5 |
| **Reaction volume** | **58.5** |

- 1. Transfer 23.5 µL of NEB End Repair Premix into wells of a destination plate.
  2. Transfer 35 µL of ss-cDNA to End Repair Premix, mix using 80% volume, 10X.
  3. Tetrad Program: LIBCOR>ER 20C, 30 min; hold 4C
  4. Safe stopping point if stored at -20C.

1. **Bead Clean End Repaired & Phosphorylated Template**
   1. Ethanol and Magnetic beads must be incubated at room temperature for at least 30 minutes before use.

| **DNA volume (µL)** | **Bead Volume (µL)** | **Mixing Volume (µL)** | **Bead Binding Time (mins)** | **Magnet Clearing Time (mins)** | **Supernatant Volume (µL)** | **2x 70% EtOH Wash Vol (µL)** | **Ethanol Air Dry Time (mins)** | **EB Elution Volume (µL)** | **Elution Time (mins)** | **Magnet Elution Time (mins)** | **Transfer Volume (µL)** |
| --- | --- | --- | --- | --- | --- | --- | --- | --- | --- | --- | --- |
| 58.5 | 105 | 131 | 15 | 7 | 163.5 | 150 | 5 | 32 | 3 | 2 | 30 |

- 1. Note: This is a safe stopping point. Do not proceed to adenylation unless you have adequate time to perform ligation reaction as well.

1. **A-Tailing**

| **Solution** | **1 rxn (µL)** |
| --- | --- |
| End-Repair + BC DNA | 30 |
| NEB Adenylation Premix | 20 |
| **Reaction volume** | **50** |

- 1. Transfer 20 µL of NEB Adenylation Premix to 30µL of size selected and repaired/phosphorylated DNA.
  2. Tetrad Program: LIBCOR>ATAIL
  3. Proceed directly to in-tandem ligation (do not bead clean after Adenylation). Store on ice while preparing Ligation premix and adapters.

1. **Adapter Ligation**

Ligation_Brew_4 pmoles:

| **Solution** | **1 rxn (µL)** |
| --- | --- |
| **Adenylated template** | **50** |
| **NEB Ultra Premix 2X** | 21  Ligation_Brew_4pmol  (25µL) |
| PE Adapter (10 µM) | 0.4 |
| dH2O | 3.6 |
| **Reaction volume** | **75** |

- 1. Transfer 25µL of ligation brew to 50µL of adenylated template.
  2. Reset pipette to 80% total volume, mix 10X.
  3. Select tetrad program: LIBCOR>LIGATION
  4. Set a timer for 15 minutes. Quick spin plate and store on ice immediately after the 15 minute ligation.

1. **Double Bead Clean post Ligation**

**Bead clean #1**

| **DNA volume (µL)** | **Bead Volume (µL)** | **Mixing Volume (µL)** | **Bead Binding Time (mins)** | **Magnet Clearing Time (mins)** | **Supernatant Volume (µL)** | **2x 70% EtOH Wash Vol (µL)** | **Ethanol Air Dry Time (mins)** | **EB Elution Volume (µL)** | **Elution Time (mins)** | **Magnet Elution Time (mins)** | **Transfer Volume (µL)** |
| --- | --- | --- | --- | --- | --- | --- | --- | --- | --- | --- | --- |
| 75 | 75 | 120 | 15 | 7 | 150 | 150 | 5 | 52 | 3 | 2 | 50 |

**Bead clean #2**

| **DNA volume (µL)** | **Bead Volume (µL)** | **Mixing Volume (µL)** | **Bead Binding Time (mins)** | **Magnet Clearing Time (mins)** | **Supernatant Volume (µL)** | **2x 70% EtOH Wash Vol (µL)** | **Ethanol Air Dry Time (mins)** | **EB Elution Volume (µL)** | **Elution Time (mins)** | **Magnet Elution Time (mins)** | **Transfer Volume (µL)** |
| --- | --- | --- | --- | --- | --- | --- | --- | --- | --- | --- | --- |
| 50 | 50 | 80 | 15 | 7 | 100 | 150 | 5 | 20 | 3 | 2 | 19 |

- 1. The ligated template can be stored at -20C after the first or second bead clean up step.

1. **PCR enrich adapter-ligated template**
   1. The volume requirement for 1 reaction set up for ss-cDNA is as follows:

| **Solution** | **1 rxn (µL)** |
| --- | --- |
| Adapter Ligated + BC DNA | 19 |
| 5X Phusion HF Buffer | 10 |
| 10mM dNTP | 1  PCR Brew + USER  (27µL) |
| DMSO | 1.5 |
| Hot Start Phusion (2 U/µL) | 0.5 |
| PE PCR primer 1.0 (25 µM) | 2 |
| USER enzyme | 3 |
| dH_2_O | 9 |
| Indexed PCR primer plate (12.5µM) | 4 |
| **Reaction volume** | **50** |

- 1. Select Pipeline-specific tetrad program:

| **Starting Material** | **PCR cycles** | **Tetrad Program** |
| --- | --- | --- |
| ss-cDNA(poly-A/ribo-depleted) | 13 | SSCDNA13 |

- 1. PCR-enriched template can be stored at -20C or proceed immediately to bead clean PCR enriched template.

1. **Double Bead Clean post iPCR**

**Bead clean #1**

| **DNA volume (µL)** | **Bead Volume (µL)** | **Mixing Volume (µL)** | **Bead Binding Time (mins)** | **Magnet Clearing Time (mins)** | **Supernatant Volume (µL)** | **2x 70% EtOH Wash Vol (µL)** | **Ethanol Air Dry Time (mins)** | **EB Elution Volume (µL)** | **Elution Time (mins)** | **Magnet Elution Time (mins)** | **Transfer Volume (µL)** |
| --- | --- | --- | --- | --- | --- | --- | --- | --- | --- | --- | --- |
| 50 | 50 | 80 | 15 | 7 | 100 | 150 | 5 | 52 | 3 | 2 | 50 |

**Bead clean #2**

| **DNA volume (µL)** | **Bead Volume (µL)** | **Mixing Volume (µL)** | **Bead Binding Time (mins)** | **Magnet Clearing Time (mins)** | **Supernatant Volume (µL)** | **2x 70% EtOH Wash Vol (µL)** | **Ethanol Air Dry Time (mins)** | **EB Elution Volume (µL)** | **Elution Time (mins)** | **Magnet Elution Time (mins)** | **Transfer Volume (µL)** |
| --- | --- | --- | --- | --- | --- | --- | --- | --- | --- | --- | --- |
| 50 | 50 | 80 | 15 | 7 | 100 | 150 | 5 | 25 | 3 | 2 | 23 |

- 1. Template can be stored at -20C after the first or second bead clean up post PCR.

1. **QC Final Library Products**
   1. Run 1uL of each final library product on Agilent DNA 1000 chip assay.
   2. Quantify each final library product by Qubit HS DNA assay.
   3. If required, pool samples by equal molar pooling and quantify pool by Qubit HS DNA assay.
