# Supplementary figures and images for: Increasing quality, throughput and speed of sample preparation for strand-specific messenger RNA sequencing
Source: BMC Genomics. 2017 Jul 5;18:515. doi: 10.1186/s12864-017-3900-6 (PMC5499059; doi:10.1186/s12864-017-3900-6)

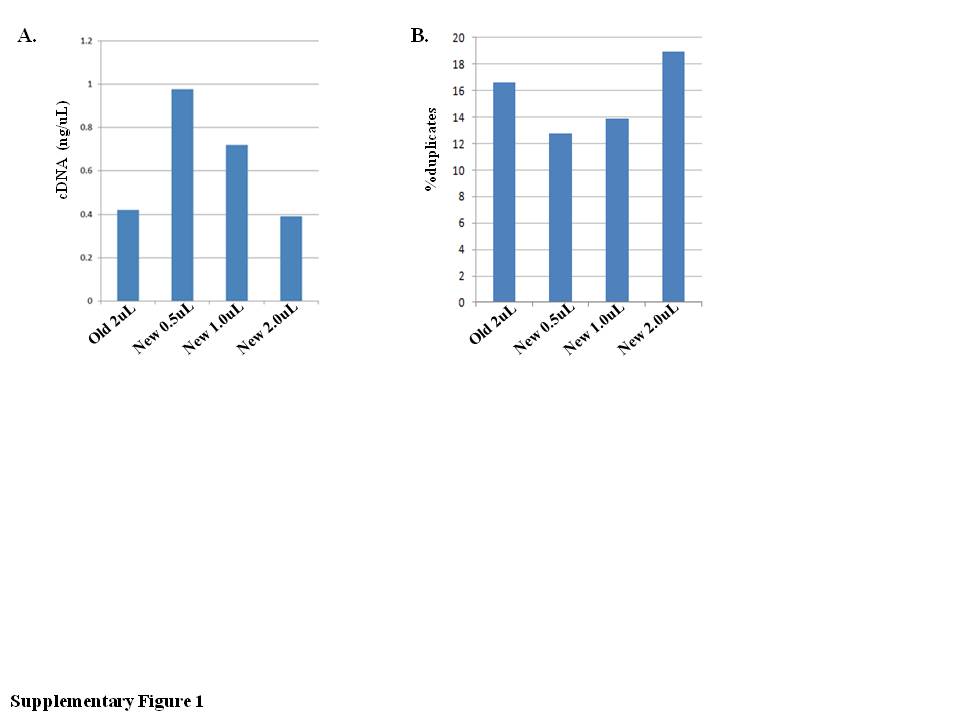

Supplement: Supplementary file 4 — Superscript II titration. 3.5 μg UHR was used as input Total RNA followed by multiMACS mRNA isolation. Various amounts of Superscript II were used in a 25 μL total reaction volume of 1st strand synthesis. “New” and “old” denote two different lots of the enzyme. (A) cDNA yield. (B) Duplicate rate. Strand-specific libraries were generated from the cDNA samples shown in (A). Libraries were pooled and sequenced using PE75. 20 million reads from each of the libraries were sampled for calculating duplicate rate. (JPEG 31 kb) [file 12864_2017_3900_MOESM4_ESM.jpg]

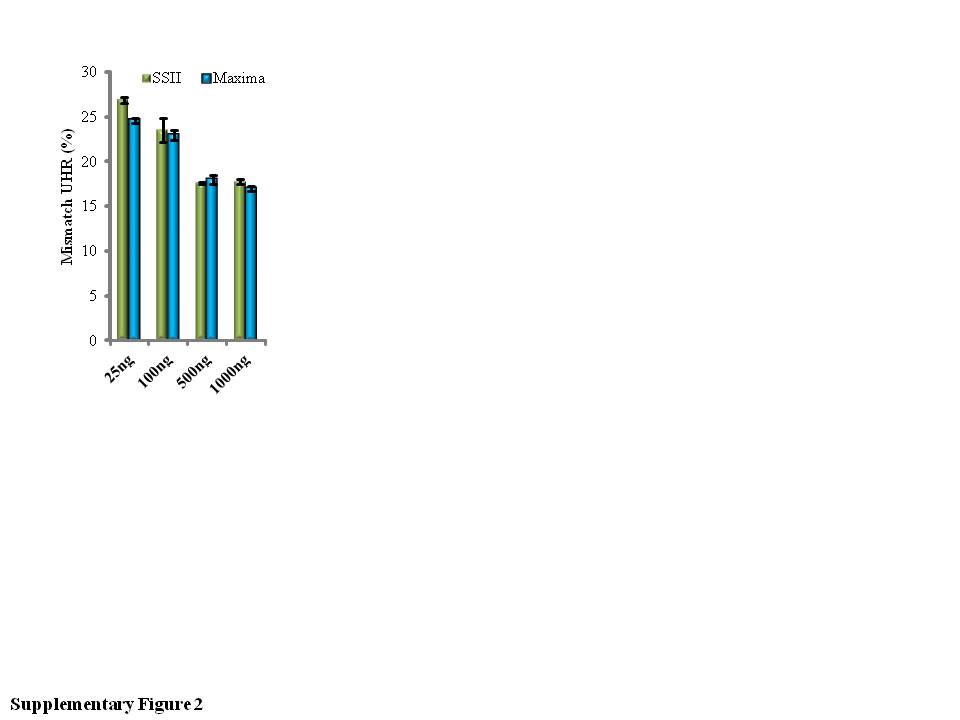

Supplement: Supplementary file 6 — Reverse transcriptases and UHR sequence divergence. UHR whole transcriptome data was assessed for divergence of sequence relative to a compendium of human reference comprised of known single nucleotide polymorphisms (SNPs). n = 3; error bars = Standard Deviation. (JPEG 24 kb) [file 12864_2017_3900_MOESM6_ESM.jpg]

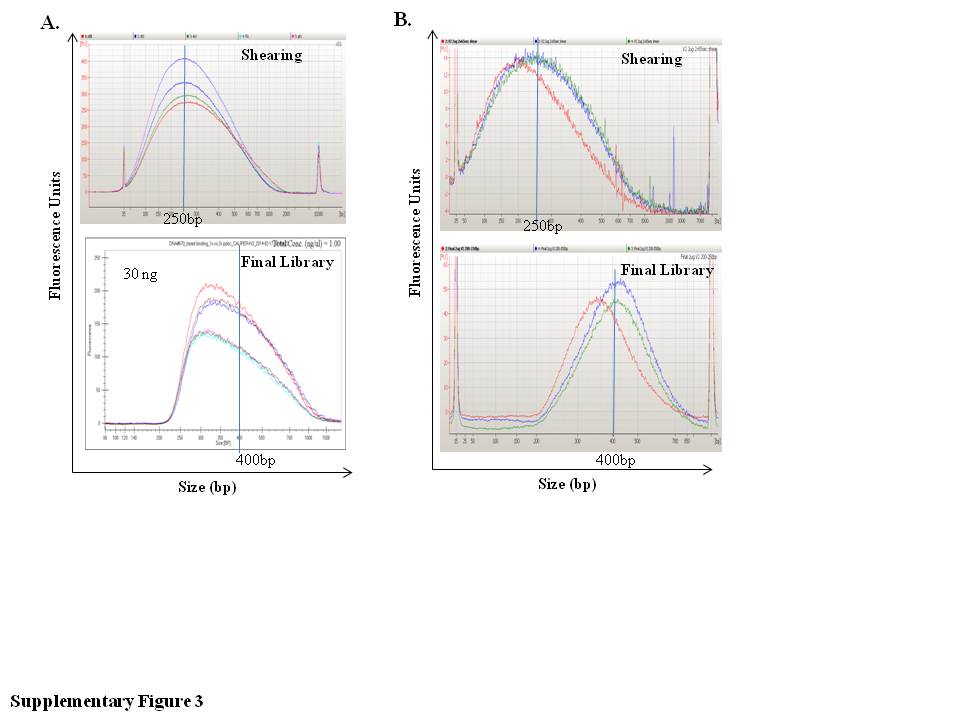

Supplement: Supplementary file 7 — Larger strand-specific library sizes are generated using 1:1 post-ligation and post- ﻿UNG bead-based purifications. (A) gDNA libraries. Upper panel is a trace representing size profile of sheared HL60 gDNA (replicates are overlayed). 30 ng was used for 200 nt-gap library construction where post-ligation clean up was performed twice with 1:1 ratio of bead volume to DNA volume. Lower panel is size profile of final libraries. (B) Strand-Specific cDNA libraries. Upper panel is a trace representing size profile of sheared double-strand cDNA. cDNA was generated from 2 μg UHR t﻿otal RNA followed by multiMACS mRNA isolation. Post-ligation clean up was performed once with 1:1. Post-UNG was also once with 1:1. Lower panel is size profile of final libraries. Note that the strand-specific cDNA libraries are significantly larger than the gDNA libraries despite the sheared starting material being of similar size profile. (JPEG 50 kb) [file 12864_2017_3900_MOESM7_ESM.jpg]

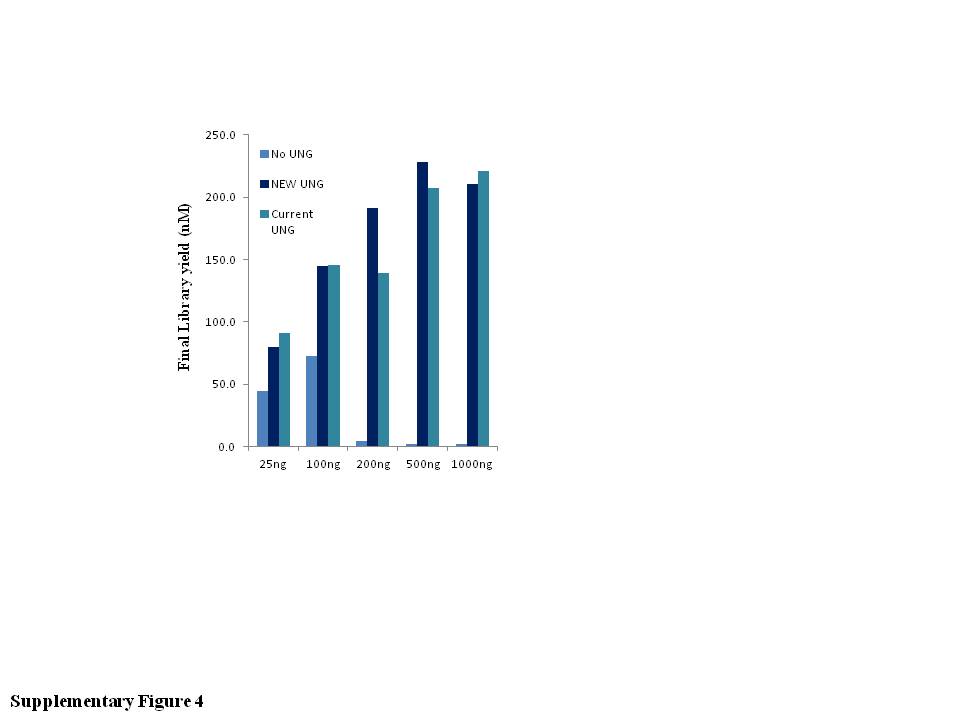

Supplement: Supplementary file 8 — UNG digestion improves library yield. Libraries were made from the indicated UHR input amounts in the presence or absence of UNG in the PCR reaction. UNG from two different vendors was used. n = 2. (JPEG 26 kb) [file 12864_2017_3900_MOESM8_ESM.jpg]

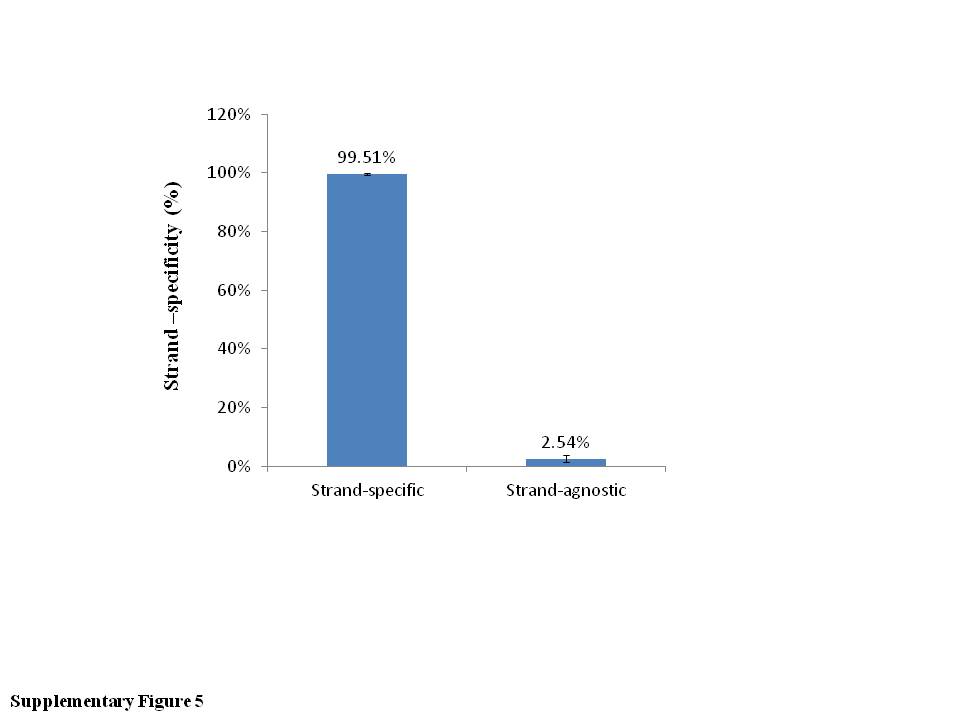

Supplement: Supplementary file 9 — The performance of our tool for calculating strand-specificity. (JPEG 23 kb) [file 12864_2017_3900_MOESM9_ESM.jpg]

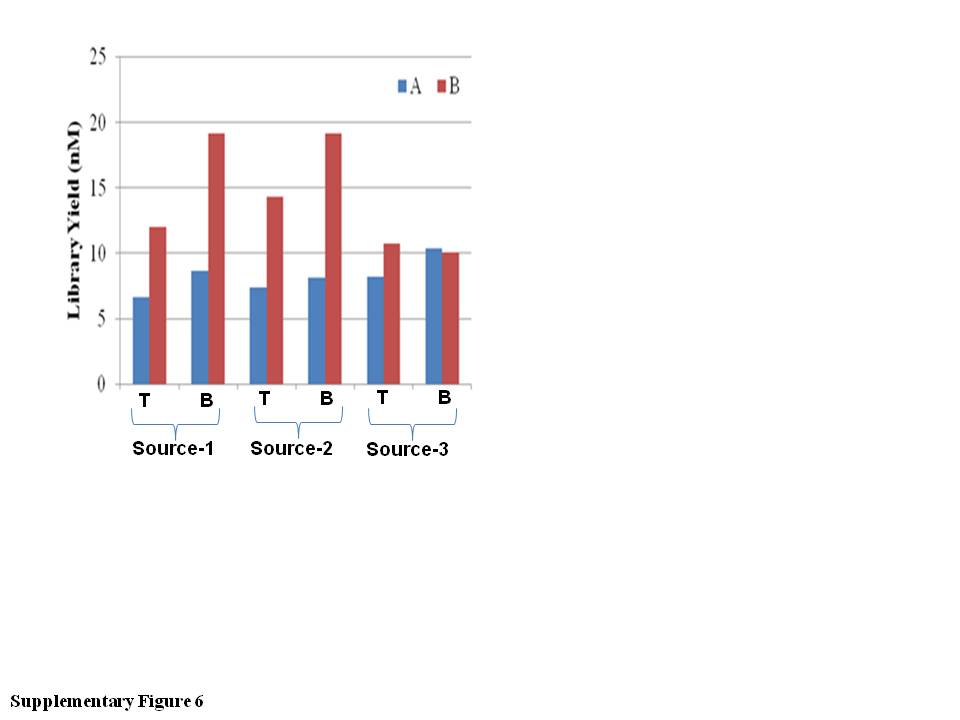

Supplement: Supplementary file 10 — Library construction chemistry comparison using tumor samples. Two NEB workflows were evaluated. The first has bead clean up after each of the library construction steps (A); the second has the bead cleanup after A-tail removed (B). Input gDNA was from three different clinical samples (Source 1–3). (JPEG 27 kb) [file 12864_2017_3900_MOESM10_ESM.jpg]

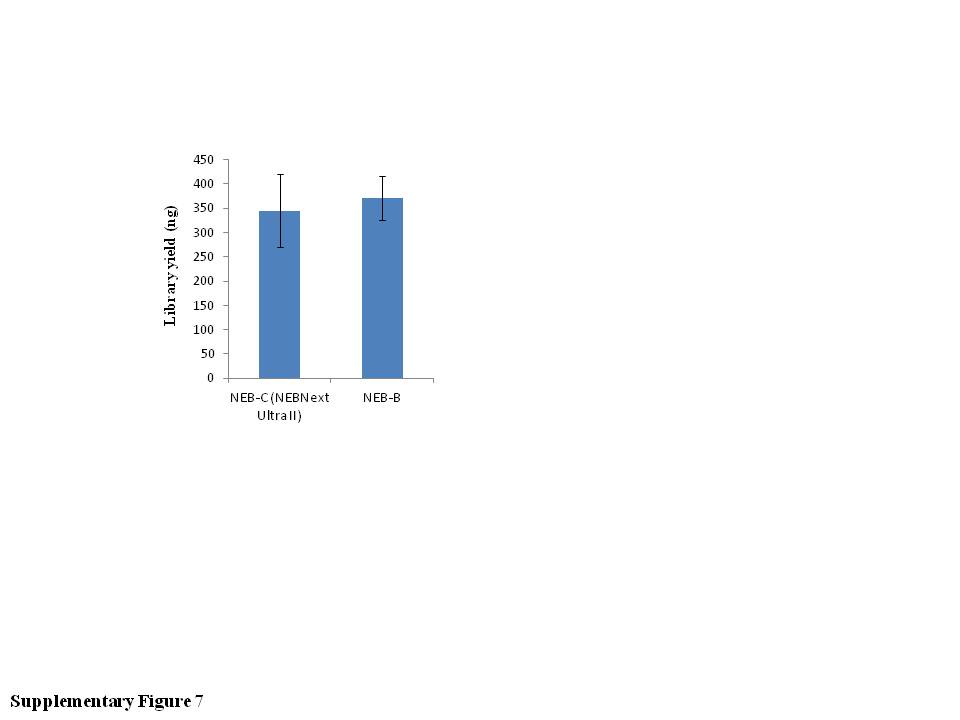

Supplement: Supplementary file 11 — Library yield is improved with the latest streamlined NEB library construction protocol (NEBUltraII). 100 ng HeLa gDNA was used as input and 6 cycles of PCR was applied. PCR module with Phusion enzyme was the same for both. NEB-B is the most optimal protocol identified as shown in Fig. 7. (JPEG 20 kb) [file 12864_2017_3900_MOESM11_ESM.jpg]

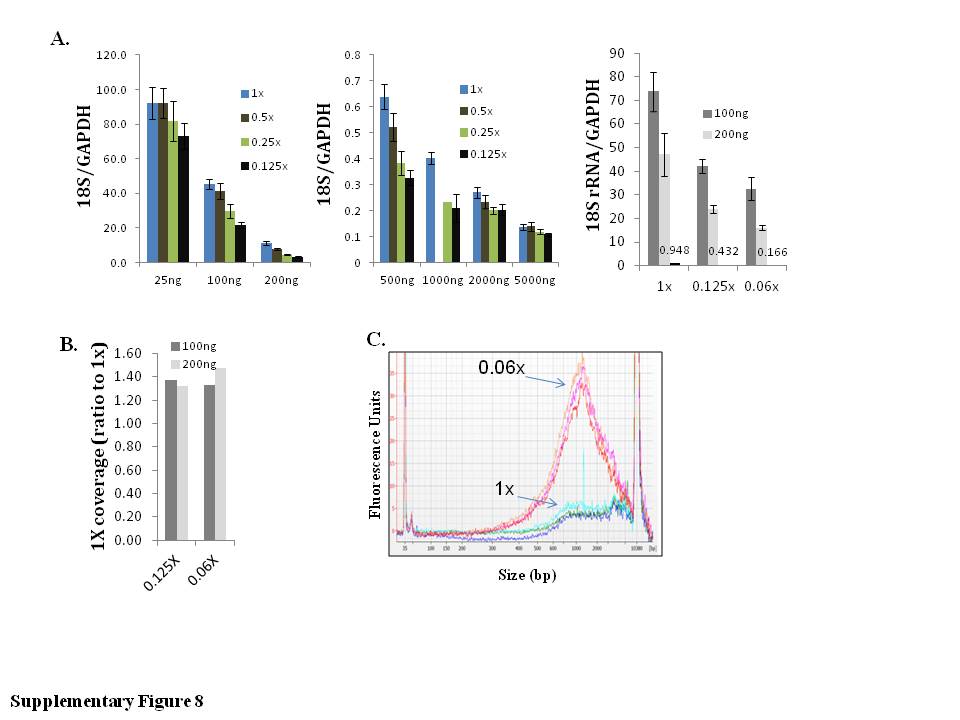

Supplement: Supplementary file 12 — Oligo-dT bead titration for MultiMACS mRNA isolation and its effect on % rRNA and mRNA/cDNA yield. (A) % rRNA as assessed by qPCR. Oligo-dT bead amount was titrated. Indicated amounts are expressed relative to manufacturer specified amount (1×). qPCR was applied to measure levels of 18S rRNA and GAPDH mRNA. The ratio of 18S to GAPDH levels was calculated based on the Pfaffl method (Pfaffl MW, 2001) and is shown graphically. (B) Duplicate rate upon sequencing. For selected bead amounts, libraries were generated and sequenced. Y-axis is ratio of duplicate rate calculated relative to the manufacturer specified amount (1×). (C). Oligo-dT bead amount and cDNA yield. Of note, this represents the latest lots of the beads which appear to have significantly underperformed where the effect of lower bead amount is most drastic. (JPEG 56 kb) [file 12864_2017_3900_MOESM12_ESM.jpg]

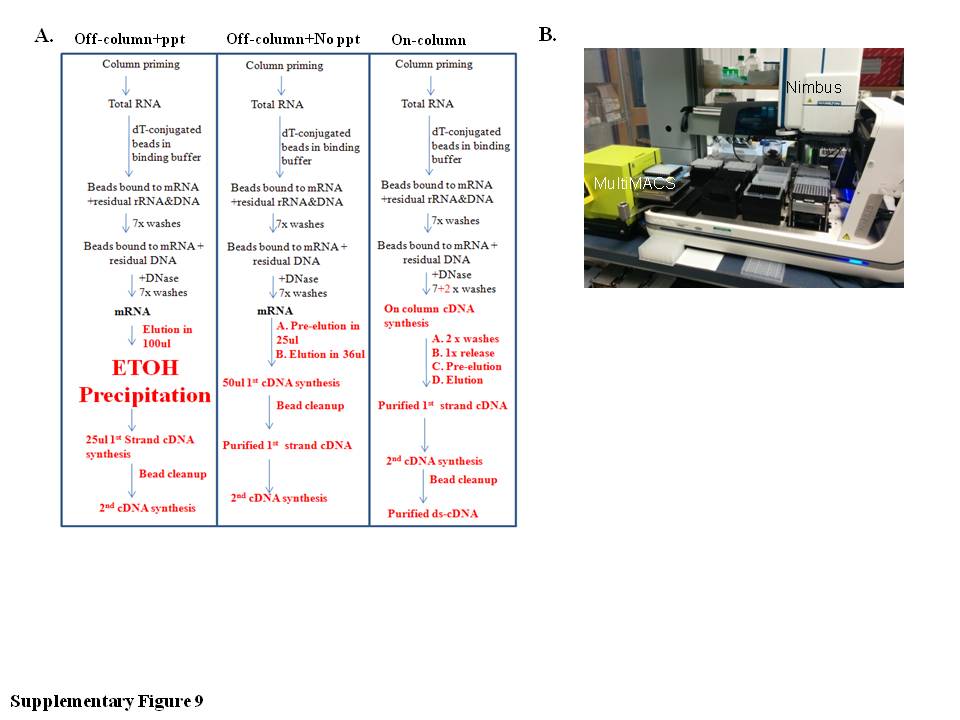

Supplement: Supplementary file 13 — Workflow and automation of various MiltiMACS mRNA isolation formats and on-column cDNA synthesis. Streamlined MultiMACS that does not require precipitation before cDNA synthesis (no ppt) versus another version that requires precipitation (ppt) as well as on-column cDNA synthesis are depicted in (A). In (B), the integration of the MutiMACS unit with Hamilton Nimbus Microlab liquid handler is shown. (JPEG 66 kb) [file 12864_2017_3900_MOESM13_ESM.jpg]

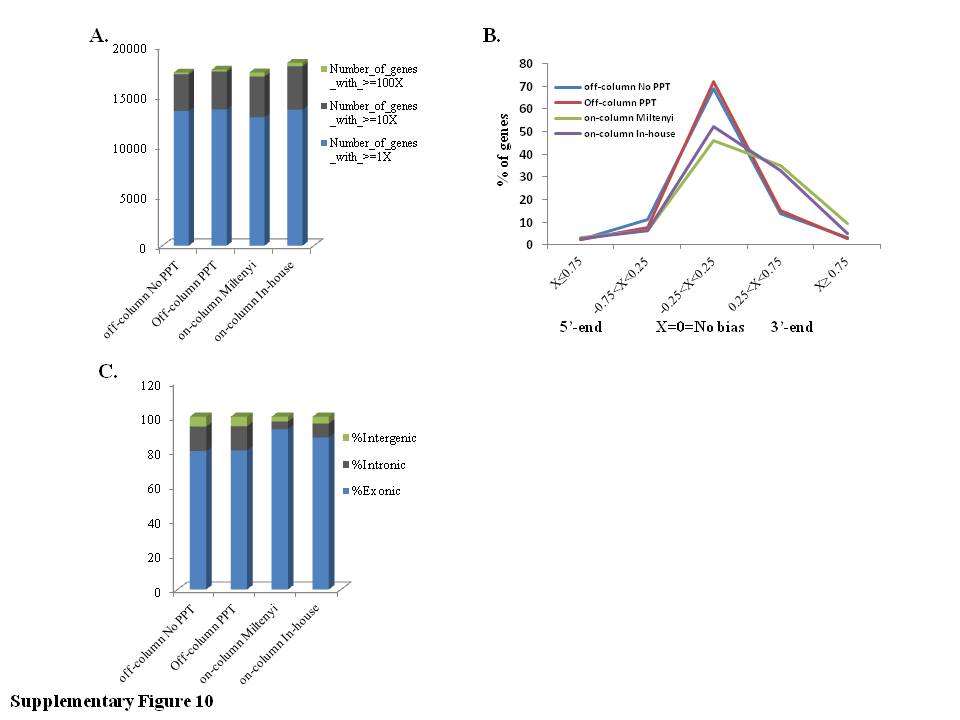

Supplement: Supplementary file 14 — Comparison of various MiltiMACS mRNA isolation formats and on-column cDNA synthesis. Streamlined MultiMACS that does not require precipitation before cDNA synthesis (no ppt) versus another version that requires precipitation (ppt) as well as on-column cDNA synthesis were compared. For the latter, two different cDNA synthesis mixes were used: one that comes with the Miltenyi kit that involves dT-priming and another that is based on our protocol which is spiked with random hexameres (in-house). (A) Detection of gene expression. Number of genes whose expression is detected at various degrees of coverage is shown for all the four conditions. (B) 3′-end bias. X-axis values represent proportion of genes whose coverage shows a certain degree of asymmetry. When the proportion with asymmetric coverage (x) is 0, there is no bias at all. When x > 0, there more bias towards the 3′-end and vice versa. (C) Mapping to various transcriptomic regions. n = 3. (JPEG 49 kb) [file 12864_2017_3900_MOESM14_ESM.jpg]

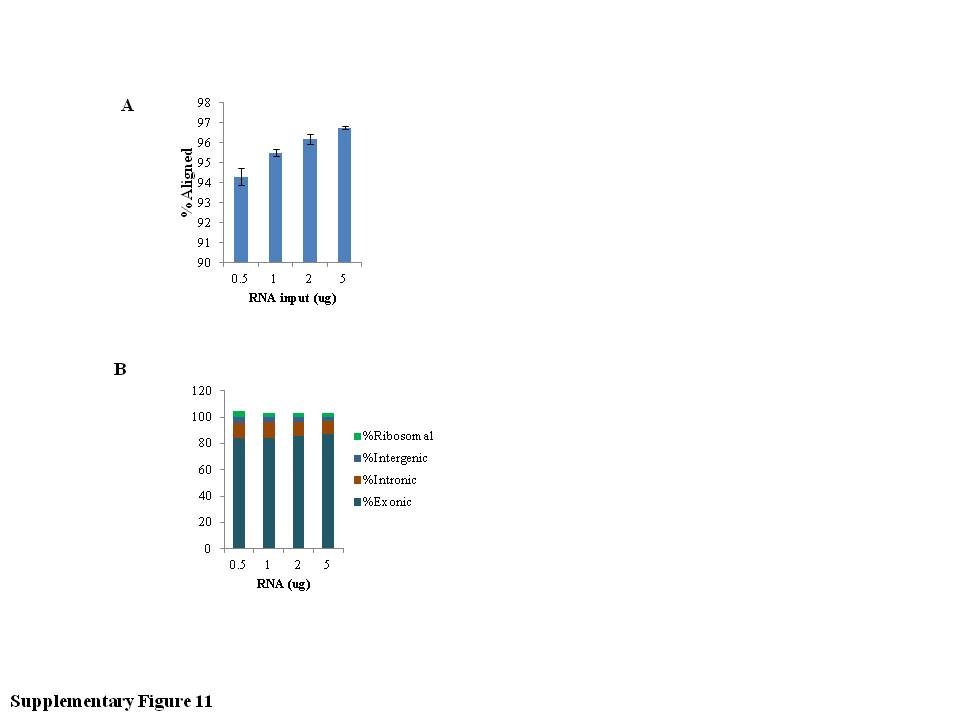

Supplement: Supplementary file 15 — Total RNA input titration using the intermediate ssRNA-seq pipeline. The protocol evaluated includes all changes (1st strand cDNA synthesis, optimal bead purifications, new library construction chemistry with modified ligation condition, bead-based size selection, and UNG treatment) with the exception of the mRNA isolation improvements. (A) Comparable mapping of reads to the human genome. (B) Comparable mapping of reads to various transcriptome catagories. (C) High correlation of expression between lower input and higher input libraries. Pearson’s correlation coefficient was calculated pair-wise as indicated. Heat map was generated for the resulting values with color intensity representing the degree of correlation as per the depicted legend. n = 3 for all inputs except for 2μg and 5μg input amounts where only duplicates were represented. Error bars = Standard Deviation. (JPEG 27 kb) [file 12864_2017_3900_MOESM15_ESM.jpg]

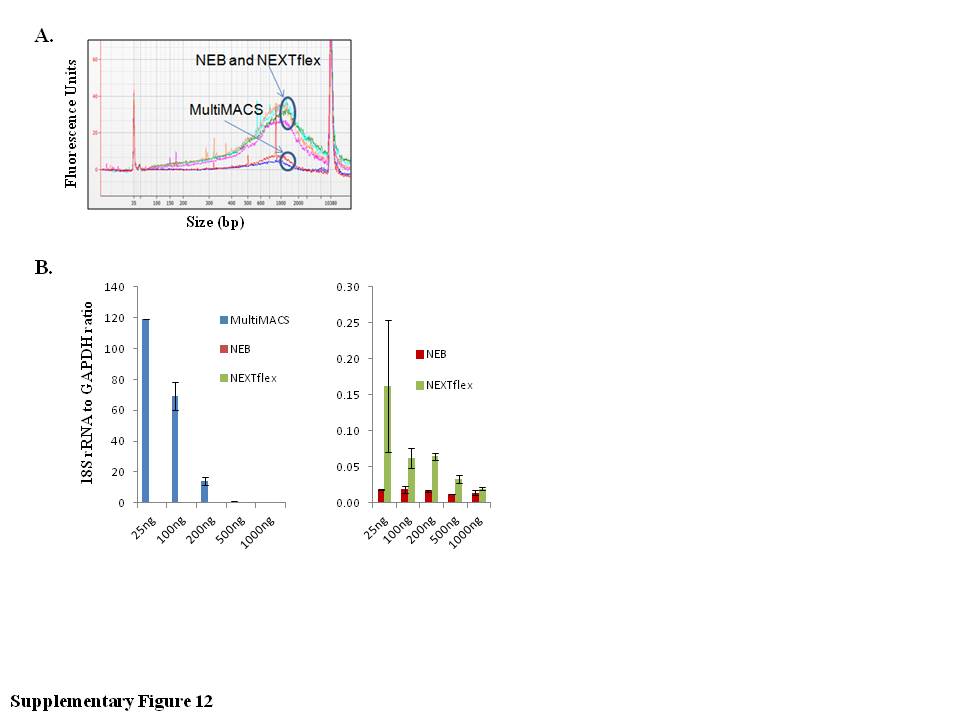

Supplement: Supplementary file 16 — Comparison of mRNA isolation kits. The modified MultiMACS protocol with 1/16th bead amount was compared to two other kits (NEXTflex and NEB). Various UHR inputs were used. (A) cDNA yield. (B) % rRNA as assessed by qPCR. Details are as in Additional file 12: Figure S8. All three are shown in the left panel. Right panel shows just the NEXTflex and NEB on a different scale. (JPEG 37 kb) [file 12864_2017_3900_MOESM16_ESM.jpg]

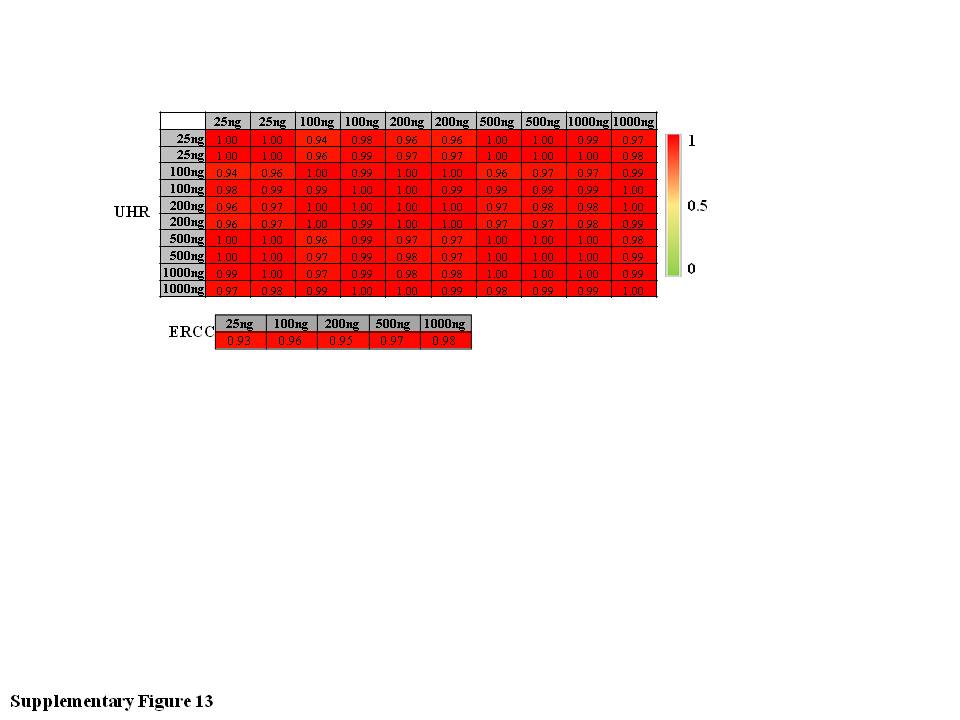

Supplement: Supplementary file 17 — High correlation of expression between lower input and higher input libraries. Upper panel is for UHR data. Lower panel: High correlation of observed versus expected levels of ERCC spike-in synthetic RNAs. Pearson’s correlation coefficient was calculated pair-wise among the libraries (UHR data) and between what was measured in the libraries versus theoretically expected levels for the ERCC RNAs. n = 2. (JPEG 50 kb) [file 12864_2017_3900_MOESM17_ESM.jpg]

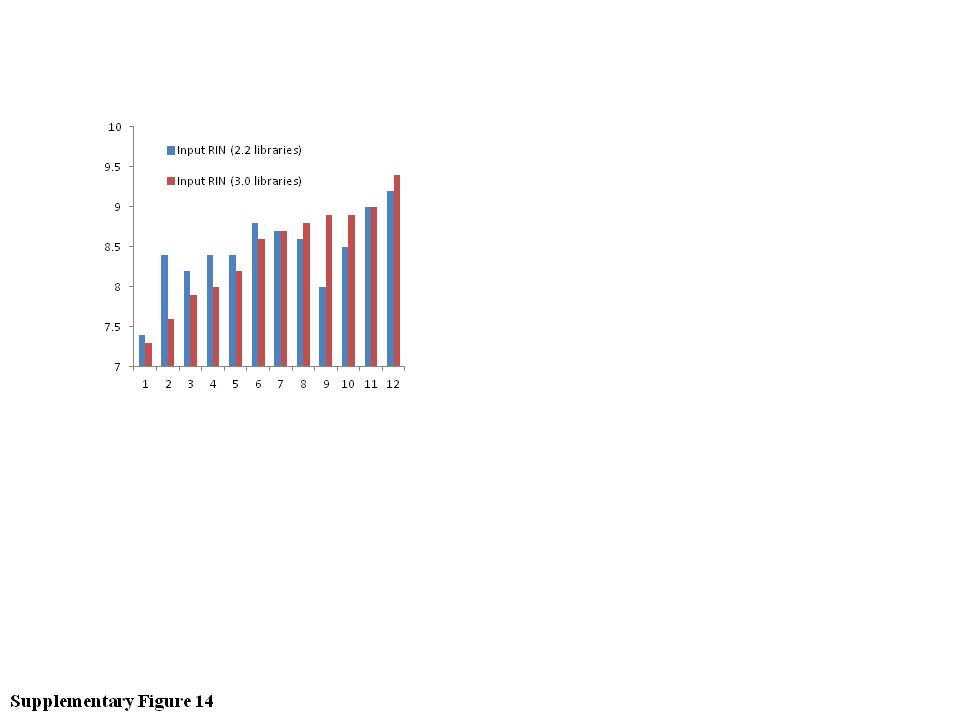

Supplement: Supplementary file 18 — RNA integrity comparisons between the input RNAs for the previous and newer libraries. RNA integrity (RIN) is based on Agilent RNA Nano assay. (JPEG 25 kb) [file 12864_2017_3900_MOESM18_ESM.jpg]

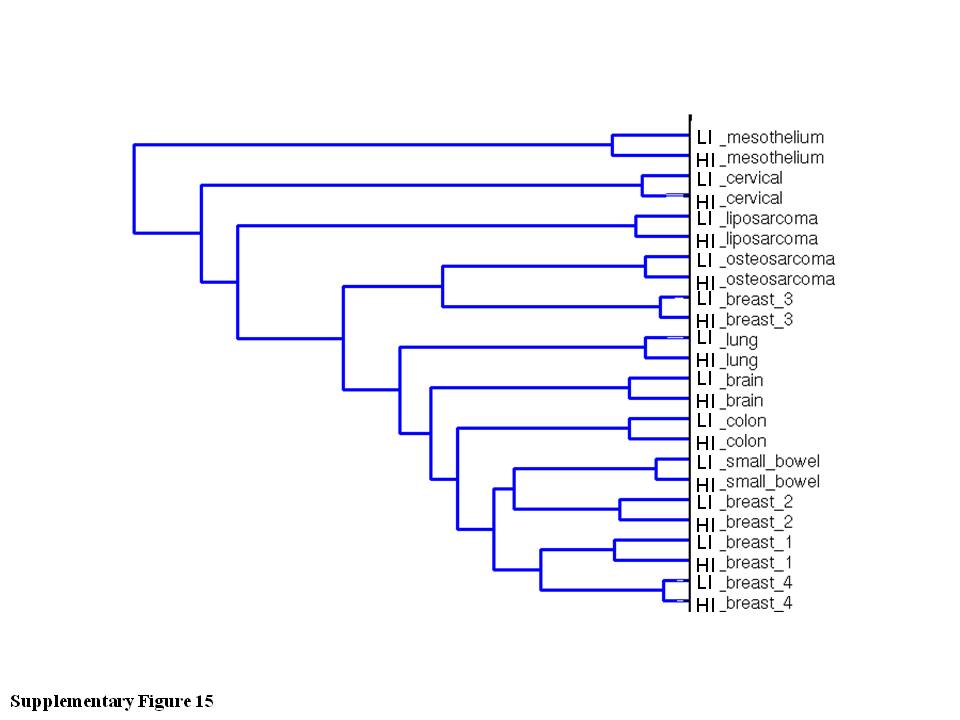

Supplement: Supplementary file 19 — Hierarchical clustering data that shows segregation of clades in a sample dependent manner as opposed to segregation by input amount RNA. Samples are as in Fig. 9. LI = low input; HI = high input. (JPEG 50 kb) [file 12864_2017_3900_MOESM19_ESM.jpg]
